# Investigation of the milling characteristics of different focused-ion-beam sources assessed by three-dimensional electron diffraction from crystal lamellae

**DOI:** 10.1107/S2052252523001902

**Published:** 2023-03-24

**Authors:** James M. Parkhurst, Adam D. Crawshaw, C. Alistair Siebert, Maud Dumoux, C. David Owen, Pedro Nunes, David Waterman, Thomas Glen, David I. Stuart, James H. Naismith, Gwyndaf Evans

**Affiliations:** a Rosalind Franklin Insititute, Harwell Science and Innovation Campus, Didcot, Oxford OX11 0QX, United Kingdom; b Diamond Light Source, Harwell Science and Innovation Campus, Didcot, Oxford OX11 0QS, United Kingdom; c Research Complex at Harwell, Harwell Science and Innovation Campus, Harwell, Oxford OX11 0FA, United Kingdom; d CCP4, Harwell Science and Innovation Campus, Didcot, Oxford OX11 0FA, United Kingdom; e Division of Structural Biology, University of Oxford, Roosevelt Drive, Oxford OX3 7BN, United Kingdom; University of California, Los Angeles, USA

**Keywords:** pFIB milling, crystal lamellae, 3DED, beam damage, 3D electron diffraction

## Abstract

Three-dimensional electron diffraction (3DED) from nanocrystals of biological macromolecules requires the use of very small crystals. These are typically less than 300 nm-thick in the direction of the electron beam due to the strong interaction between electrons and matter. In recent years, focused-ion-beam (FIB) milling has been used in the preparation of thin samples for 3DED. These instruments typically use a gallium liquid metal ion source. Inductively coupled plasma (ICP) sources in principle offer faster milling rates. Little work has been done to quantify the damage these sources cause to delicate biological samples at cryogenic temperatures. Here, an analysis of the effect that milling with plasma FIB (pFIB) instrumentation has on lysozyme crystals is presented. This work evaluates both argon and xenon plasmas and compares them with crystals milled with a gallium source. A milling protocol was employed that utilizes an overtilt to produce wedge-shaped lamellae with a shallow thickness gradient which yielded very thin crystalline samples. 3DED data were then acquired and standard data-processing statistics were employed to assess the quality of the diffraction data. An upper bound to the depth of the pFIB-milling damage layer of between 42.5 and 50 nm is reported, corresponding to half the thickness of the thinnest lamellae that resulted in usable diffraction data. A lower bound of between 32.5 and 40 nm is also reported, based on a literature survey of the minimum amount of diffracting material required for 3DED.

## Abstract 2

An analysis of the effect on diffraction quality that arises from milling crystals of lysozyme with argon and xenon plasma focused ion beams is presented. This is compared with crystals milled using a gallium source. Upper and lower bounds for the thickness of the milling damage layer are estimated.

## 1. Introduction

Three-dimensional electron diffraction (3DED) of nanocrystals of biological macromolecules (Gemmi *et al.*, 2019 ▸) using the rotation method of data collection and processing (Arndt & Wonacott, 1977 ▸) has become an established technique in recent years (Wan *et al.*, 2013 ▸; Nannenga *et al.*, 2014 ▸ *b*; Clabbers *et al.*, 2017 ▸; Beale *et al.*, 2020 ▸). 3DED has been proposed as an umbrella term for the various techniques that perform ED on 3D crystals (Gemmi *et al.*, 2019 ▸; Gruene & Mugnaioli, 2021 ▸). Other common names used to refer to similar techniques include automated diffraction tomography, rotation electron diffraction, continuous rotation electron diffraction and micro-crystal electron diffraction, amongst others. 3DED We differentiate 3DED from the well established method of 2D electron crystallography which produces high-resolution structures of proteins but uses a different mode of data collection and processing (Unwin & Henderson, 1975 ▸; Henderson *et al.*, 1990 ▸; Unwin, 1995 ▸). Simulations of the diffracted intensities from crystals 1–5 unit cells-thick show significant differences to the diffracted intensities from a 3D crystal 50 unit cells-thick (Gorelik *et al.*, 2021 ▸). Thus, it is possible to distinguish experimentally between diffraction resulting from a 2D crystal as opposed to a 3D crystal. Experimental studies have shown that it is possible to solve the structure of catalase using 3DED from single nanocrystals with an estimated thickness of 70 to 150 nm, corresponding to crystals 5–10 unit cells-thick (Yonekura *et al.*, 2015 ▸; Nannenga *et al.*, 2014 ▸ *a*). This experimental and theoretical evidence suggests that the lower bound on the sample thickness required to obtain a structure solution from 3DED data is around 5 unit cells. For lysozyme, 5 unit cells along the shortest unit cell direction is ∼20 nm. Therefore, a thickness of at least 20 nm of undamaged crystal is assumed to be required.

In 3DED, the strong interaction between electrons and matter (Henderson, 1995 ▸; Clabbers & Abrahams, 2018 ▸) requires samples of typically less than 300 nm since the mean free path of an electron in vitreous ice is around 300 nm for electrons accelerated at 300 keV in a transmission electron microscope (TEM). In recent years, focused-ion-beam (FIB) milling has been used in the preparation of electron-transparent samples suitable for ED (Duyvesteyn *et al.*, 2018 ▸; Li *et al.*, 2018 ▸; Zhou *et al.*, 2019 ▸; Martynowycz *et al.*, 2019 ▸ *b*; Beale *et al.*, 2020 ▸) as well as for cryo-electron tomography, cryo-ET (Harapin *et al.*, 2015 ▸; Schaffer *et al.*, 2017 ▸). Using this approach, a thick specimen can be progressively milled to produce a lamella of less than 300 nm-thick for imaging or diffraction in a TEM.

Previous studies have utilized gallium ion beams to mill crystals (Duyvesteyn *et al.*, 2018 ▸; Martynowycz *et al.*, 2019 ▸ *a*; Beale *et al.*, 2020 ▸; Martynowycz *et al.*, 2021 ▸). The availability of inductively coupled plasma (ICP) sources for milling instruments potentially offers an increase in milling throughput via access to higher beam currents than are possible with gallium beams (Smith *et al.*, 2006 ▸). Although there is considerable experience in using gallium for milling, little has been reported on the extent of damage that ICP sources have on biological samples at cryogenic temperatures. Such an analysis is critical if such instruments are to be deployed (Dumoux *et al.*, 2022 ▸; Berger *et al.*, 2022 ▸). Studies on hard materials have demonstrated that xenon plasma beams produce less damage than gallium at a given current and allow a higher milling rate (Burnett *et al.*, 2016 ▸). Compared with gallium sources used at the same energies, milling single-crystal silicon with a xenon source has been shown to result in less sidewall amorphization damage (Kelley *et al.*, 2013 ▸). Investigations using metal alloys have also shown that xenon-milling produces a smaller damaged zone immediately beneath the amorphization layer (Liu *et al.*, 2020 ▸). In materials science, high-resolution information can be measured directly; however, the damage sensitivity of frozen hydrated samples does not allow atomic observation and high-resolution information is obtained using averaging methods (Scheres, 2012 ▸; Wan & Briggs, 2016 ▸), preventing any direct observation. Therefore, it is not possible to directly observe beam-induced damage. However, for cryogenic biological samples, it has been observed that electron beam damage leads to loss of high spatial frequencies from multifactorial and concomitant events such as local devitrification, local mechanical distortion, ion implantation and chemical interaction. This loss of high spatial frequencies can be conveniently observed from ED data collected from single-crystal lamellae of proteins. Using 3DED to perform this analysis has the advantage that protein crystals, owing to their long-range order and fragility, are extremely sensitive to damage and it is therefore possible to infer the depth of the damage layer by observing the quality of diffraction as a function of lamella thickness, thereby distinguishing between the relative thicknesses of ordered (undamaged) and disordered (damaged) material.

Here we present an analysis of the damage from a pFIB using 3DED data from lamellae of crystalline lysozyme as a pr­oxy. The composition of protein crystals is a homogeneous representation of biological samples and produces clear diffraction patterns. The introduction of lattice disorder due to damage will result in a loss of information. Diffraction from non-milled wedge-shaped lysozyme crystals 100–600 nm-thick has previously been reported (Nannenga *et al.*, 2014 ▸ *b*) and a study of gallium-milled lamellae of various thicknesses showed no decrease in diffraction quality down to a targeted thickness of 95 nm at an electron energy of 200 keV (Martynowycz *et al.*, 2021 ▸). We fabricated wedge lamellae with a shallow thickness gradient along their length which tapered towards zero at the thin end of each lamella. This allowed data to be collected from very thin crystalline samples. We used an ICP source with argon or xenon and a gallium liquid metal ion source. We acquired 3DED data and used standard data-processing statistics to assess the quality. Some recent work has been done to qualitatively compare ED data quality from different milling sources (Martynowycz *et al.*, 2022 ▸). We collected a larger number of ED datasets from samples prepared using each milling source and included thickness measurements of the lamellae which are critical in systematically comparing the quality of the diffraction data. We use this information to infer the limits of the depth of the milling damage layer that results from pFIB, with an upper bound to the damage layer of 50, 45 and 42.5 nm, for argon, xenon and gallium, respectively, and a lower bound of 40, 35 and 32.5 nm, for argon, xenon and gallium, respectively.

## 2. Methods

Lysozyme crystals were prepared on cryo-EM grids and milled using a cryo-FIB instrument. The milled lamellae were then transferred to a TEM for collection of 3DED data. After diffraction data collection was performed, the lamellae were transferred to a TEM equipped with an energy filter to collect measurements of the sample thickness via the log-ratio method. Finally, tomography was performed on the lamellae to validate the log-ratio thickness measurements. Each of these steps is described in more detail below. Since lamellae were milled from both the top and the bottom, any damage will be propagated from both faces of the lamella. To determine an upper bound to the depth of the damage layer, *T*_damage_, from the pFIB or gallium FIB, crystal lamellae of lysozyme were milled with a stage geometry which produced wedge-shaped tapered lamellae allowing access to extremely thin crystalline regions. Our approach rests on the assumption that, at some critical thickness, *T*_critical_, there is insufficient undamaged material for diffraction such that *T*_critical_ ≤ 2*T*_damage_.

### 2.1. Crystallization

Lysozyme crystals measuring approximately 5 × 3 × 3 µm were prepared by a previously described batch method (Martin-Garcia *et al.*, 2017 ▸) which uses temperature variation to produce crystals of consistent size and morphology. To produce lysozyme crystals of the necessary size, the crystallization was performed at 24°C.

### 2.2. Grid preparation

The crystal slurry was diluted to a final concentration of 10% *v*/*v* and applied to UltrAuFoil Gold 200 mesh 2/2 cryo-EM grids (Storm *et al.*, 2020 ▸). Prior to loading in the FIB-SEM, the grids were mounted in specific FIB-compatible AutoGrid rings and secured with a c-clip (Thermo Fisher Scientific).

### 2.3. Cryo-FIB-milling of wedge-shaped crystal lamellae

The grids were loaded into a Helios Hydra G4 Dual Beam pFIB-SEM (Thermo Fisher Scientific). Alternatively, a Scios Dual Beam FIB-SEM was used (Thermo Fisher Scientific) as previously described (Duyvesteyn *et al.*, 2018 ▸; Beale *et al.*, 2020 ▸). Both systems have a cryo-stage and cryo-shield held at approximately −190°C. The grids milled in the pFIB-SEM were initially mapped using the SEM *Maps* software (Thermo Fisher Scientific) with a 2 kV accelerating voltage and a 13 pA probe current at 800× magnification. Prior to milling, the whole grid was coated in an organoplatinum layer using the gas injection system (GIS) for 35 (pFIB-SEM) or 4 s (FIB-SEM). The thickness of the GIS layer from the pFIB-SEM has been estimated to be between 1 and 2 µm (Berger *et al.*, 2022 ▸). To mitigate charging of the lamellae during milling, an additional coat of metallic platinum was sputtered onto the sample. In the pFIB, this was performed by directing the pFIB onto a platinum rod proximal to the sample. The thickness of the platinum deposition varies with the plasma species, its accelerating voltage, the current used and the sputtering time. For argon plasma at an accelerating voltage of 16 kV and beam current of 1 µA, the sputtering time was 30 s, and for xenon at an accelerating voltage of 16 kV and beam current of 0.51 µA, the sputtering time was 35 s. These values were chosen based on the empirical criteria of observed contrast in SEM and the level of reduced charging. The FIB-SEM does not have this capability, therefore samples were sputter-coated with metallic platinum during the loading procedure before GIS deposition for 60 s at 5 mA (Quorum Technologies).

**Figure 1:**
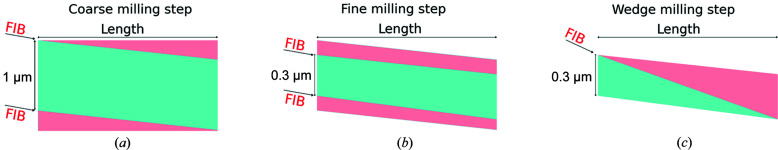
Wedge-milling protocol. (*a*) In the first step, a coarse flat lamella is progressively milled to 2 µm with a high current and then to 1 µm with a medium current. (*b*) Subsequently, a fine flat lamella is milled to 300 nm with a low current. Finally, the length of the lamella is measured and the angle with which to tilt the stage such that the wedge will taper to zero is calculated. (*c*) The wedge lamella is then milled with a low ‘polishing’ current.

Contamination-free, single, isolated crystals near the centre of the grid and at least 50 µm from a grid bar were manually selected for milling. The positions and eucentric heights of selected crystals and the desired milling locations were saved within the *Maps* software (for the pFIB-SEM) or within the *XTUI* control software (for the FIB-SEM). The contamination rate within the FIB-SEM chamber is 25 nm h^−1^, therefore the milling procedure was split into two phases. In the first phase, coarse lamellae were milled at each selected site and then, in the second phase, a final polishing step was applied to all the lamellae once all the coarse lamellae had been milled. A diagram illustrating the milling protocol is shown in Fig. 1 ▸.

For a given crystal, milling was performed in three steps, the milling spacing of each step and corresponding probe current for each source are outlined in Table 1 ▸. During the first step, narrow trenches were milled on either side of the crystal to help relieve stresses which may cause the lamella to warp or bend (Wolff *et al.*, 2019 ▸). The third and final polishing step was only performed once all the coarse-milling of lamellae had been completed across all grids. First, the lower surface of the lamella was milled, at the same milling angle as the previous coarse-milling steps. For the upper surface, the grid was tilted before milling such that, over the length of the lamella, the thickness varied from *t*_thick_ ≃ 300 nm at the edge closest to the FIB beam down to *t*_thin_ ≃ 0 at the far edge, to produce a shallow wedged lamella. The degree of tilt of the upper surface (θ) was calculated to be 1–3° using θ = *a*tan[(*t*_thick_ − *t*_thin_)/*l*_lamella_], where *l*_lamella_ is the length of the lamella. Once all the lamellae were milled, micro-sputtering of the grid was performed again to avoid beam-induced charging of the lamellae in the TEM during ED data collection. If the platinum layer is too thick, the strength of diffraction may be reduced so a short sputtering time is used at this stage. The post-milling micro-sputtering was performed for 6 (pFIB, Ar), 7.1 (pFIB, Xe) and 3 s (FIB).

### 2.4. Milled crystal lamellae

**Figure 2:**
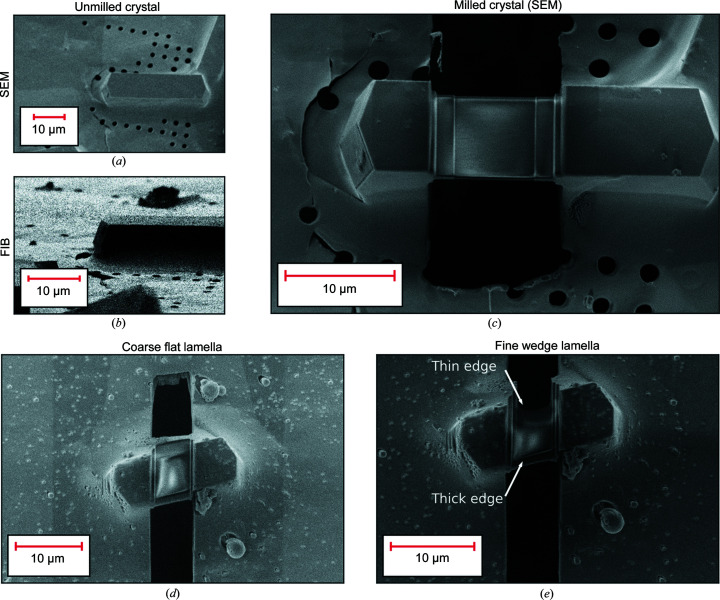
Example of a pFIB-milled crystal lamellae using the xenon plasma, unmilled in the (*a*) SEM and (*b*) FIB, and milled to a targeted thickness of 200 nm in (*c*) the SEM view. Example of a wedge lamella at (*d*) the coarse step (*e*) and final wedge step.

Fig. 2 ▸ shows examples of crystals fabricated with the pFIB both as flat and as wedge lamellae. Figs. 2 ▸(*a*) and 2 ▸(*b*) show an unmilled crystal from SEM and FIB views, respectively. For flat lamellae, as shown in Fig. 2 ▸(*c*), we targeted a typical sample thickness of 200 nm with limited curtaining. Fig. 2 ▸(*d*) shows a flat coarse-milled lamella and Fig. 2 ▸(*e*) shows a fine-milled wedge lamella. The wedge protocol enabled visual feedback because, as the sample is milled, the far edge of the lamella retreats into the crystal as the thickness reaches zero, serving as a stop signal. The average production rate of successfully milled lamellae per day was ∼16 for xenon, ∼15 for argon and ∼16 for gallium.

### 2.5. Electron diffraction data collection

3DED data were acquired with a Glacios cryo-TEM (Thermo Fisher Scientific) operated at 200 kV. Grids were carefully oriented within the Autoloader cassette to ensure that the lamella-milling direction was aligned roughly orthogonal to the microscope stage tilt axis. Data collection was performed as previously described (Shi *et al.*, 2016 ▸; Beale *et al.*, 2020 ▸) using the *SerialEM* software (Mastronarde, 2003 ▸; de la Cruz *et al.*, 2019 ▸) and a DECTRIS SINGLA detector. Preview images were saved in the MRC file format (Cheng *et al.*, 2015 ▸) and diffraction images for the rotation datasets were saved in the HDF5 file format, the native file format of the DECTRIS SINGLA detector.

Briefly, each grid was roughly aligned at the eucentric height and subsequently mapped to identify the milled lamella sites. Data collection locations were mapped and stored for batch data collection with *SerialEM*. Manual eucentric height alignment ensured the crystal remained centred in the beam. Alignments of the microscope, including the C2 aperture and diffraction beam position, were performed immediately prior to collecting diffraction data from the lamellae. The panel gap within the DECTRIS SINGLA detector necessitates the diffraction pattern be slightly offset to avoid the loss of important low-resolution data. As the DECTRIS SINGLA is radiation hard, diffraction data were measured in the absence of a beam stop meaning that the beam centre was easily determined in most cases.

Initially, ED data collection was performed in nanoprobe mode to facilitate data collection at multiple locations on the same lamella; however, we experienced practical difficulties with this approach that prevented the measurement of high-quality diffraction data, as discussed in more detail in Appendix *A* [App appa]. Consequently, all data used in this analysis were collected at a single location on the lamellae in microprobe mode. For each lamella, an arbitrary position close to the thinnest edge of the lamella was chosen to allow data collection from a range of sample thicknesses. The 50 µm C2 aperture was used with a 40 µm selected area aperture; the measured diameters of the illuminated area and selected area on the sample were ∼7 and 1.5 µm, respectively. The sample was tilted over ±40° total oscillation, with a tilt offset of 13° to correct for the milling angle of the lamella with respect to the grid, an angular range of 0.1° for each image and an exposure time of 0.2 s. The effective detector distance was 2.4 m and a spot size of 8 was used with an incident electron fluence of 0.00173 e^−^ Å^−2^ s^−1^ giving a total incident electron dose of 0.2768 e^−^ Å^−2^ per dataset. Although care was taken to ensure that, during eucentric height alignment, the data collection position remained stable at high tilt angles, since no tracking is performed during diffraction data collection, the data collection area may shift at a high tilt angle and, therefore, diffraction may come from a larger area than expected. To assess the robustness and reproducibility of the experimental setup, five datasets were collected from each lamella position. The resulting series of datasets per position were analysed to assess the diffraction behaviour as a function of dose as shown in Section S3 of the supporting information.

### 2.6. Electron diffraction data processing

The ED data were processed using the *DIALS* diffraction integration software (Winter *et al.*, 2018 ▸) using methods adapted for the processing of 3DED data (Clabbers *et al.*, 2018 ▸) as previously described (Beale *et al.*, 2020 ▸). To interpret the metadata from the *DECTRIS SINGLA* in *DIALS*, a script was written to patch the master HDF5 file, and a dedicated format class was written. A current and common issue with ED data, compared with X-ray data collected at a synchrotron, is that the image metadata may not always be accurate. Indexing 3DED data depends on accurate knowledge of the beam centre. Since the beam centre was visible on the detector, and its position was stable over the course of the collection of a single dataset, we wrote a simple script to determine the beam centre directly from the images by calculating a robust estimate of the centre of mass of the images, assuming that the direct beam is the main contributor to intensity in an image and that the beam centre is fixed throughout data collection. The reflections were indexed using the known tetragonal lysozyme space group (*P*4_3_2_1_2). However, it was common to observe that, after a single round of indexing, only a small fraction of spots were indexed. This was due to the poor estimate of the initial geometry of the beam, stage and detector. By iterating over multiple rounds of indexing and geometry refinement, it was possible to improve on the number of indexed reflections after only a few iterations to obtain an accurate estimate of the diffraction geometry and maximize the number of indexed spots.

Each ED dataset was integrated by summation and profile-fitting using the default background-modelling algorithm that considers the pixel counts to be Poisson-distributed (Parkhurst *et al.*, 2016 ▸). The data were scaled and merged using *dials.scale* (Beilsten-Edmands *et al.*, 2020 ▸). A first round of scaling was used to determine which frames should be rejected, such as any frames at high tilt angles where the diffraction may be obscured by other objects such as grid bars. The resolution of each dataset was estimated using the CC_1/2_ > 0.3 criterion (Karplus & Diederichs, 2012 ▸). A second round of scaling was then performed with a 2 Å resolution cut-off for each dataset to aid comparison of data-processing statistics between datasets. Finally, the scaled data were merged for each dataset independently and structure factors were calculated. Full data-processing parameters can be found in Section S1 of the supporting information.

### 2.7. Structure determination and refinement

A single free set of reflections for cross-validation was chosen using the *FREERFLAG* program (Winn *et al.*, 2011 ▸) and used for all datasets to ensure fair comparison. Molecular replacement with *Phaser* (McCoy *et al.*, 2007 ▸) was used to determine the phases for each scaled dataset using a known model for tetragonal lysozyme from the Protein Data Bank (PDB) as the search model (PDB entry 193L; Vaney *et al.*, 1996 ▸) determined by X-ray crystallography to a resolution of 1.33 Å. Prior to molecular replacement, the coordinates in the PDB file were randomized using *PDBSET* (Winn *et al.*, 2011 ▸) with a maximum noise level of 0.4 Å to ensure that minimal model bias was introduced. For each dataset, the structure was then refined using *REFMAC5* (Murshudov *et al.*, 2011 ▸) with electron scattering factors (Brown *et al.*, 2015 ▸). Full structure determination and refinement parameters can be found in Section S2 of the supporting information.

### 2.8. Assessment of data quality

To assess the data quality, for each dataset, standard crystallographic data-processing statistics were used. The CC_1/2_ (Karplus & Diederichs, 2012 ▸) is perhaps the most useful indicator of data quality as it provides a direct measure of the amount of information present within the scaled reflection intensities as a function of resolution. As high-resolution information is lost, the value of the CC_1/2_ tends to zero. For this reason, it is also used to derive an estimated resolution for the dataset: the reported resolution of the data is typically given at the cut-off where CC_1/2_ > 0.3. When comparing the overall CC_1/2_ between two datasets to a given resolution, higher values are preferred. The *I*/σ(*I*) and mean integrated intensity provide a measure of the strength of diffraction. The strength of diffraction depends on the amount of undamaged diffracting material. If there is more damage to the sample, the diffraction strength would be expected to be lower. The second moment of the intensities (Stein, 2007 ▸) provides a measure of the bias in the reflection intensities as a function of resolution. Deviation from the theoretical curve can indicate bias and sample pathologies in the data. The *R*_free_ and average Fourier shell correlation, FSC_average_ (Brown *et al.*, 2015 ▸) after refinement provide a measure of the quality of the refined model with a lower *R*_free_ value and a higher FSC_average_ value, indicating better agreement between model and data.

### 2.9. Lamella thickness determination via the log-ratio method

The thicknesses of the lamellae at each ED data collection position were determined using the log-ratio method (Malis *et al.*, 1988 ▸) which requires an energy filter with an energy-selecting slit to be inserted. For these measurements, the lamellae were transferred to a Krios G4 Cryo-TEM (Thermo Fisher Scientific) equipped with a Selectris X energy filter.

The *Tomo* software (Thermo Fisher Scientific) was used to map the grid and identify previously exposed areas. At each data-collection position, an image was collected with the energy-selecting slit inserted with an energy width of 10 eV, and a second image was collected with the energy-selecting slit retracted. The images were taken on a Falcon 4 detector at full readout using nanoprobe mode and a spot size of 7 at a magnification of 19500× with an exposure time of 16.5 s per image and dose rate of 5.88 e^−^ pixel^−1^ s^−1^; the pixel size was 6.3 Å, giving a dose of 2.44 e Å^−2^ for each image. The field of view was 2.58 µm and the illuminated area was 3.85 µm. The relative thickness, *t*, was then estimated according to *t* = log(*I*_t_/*I*_0_), where *I*_t_ is the total number of counts in the image with the energy filter out and *I*_0_ is the total number of counts in the image with the energy filter in. The absolute thickness in nanometres was then obtained by simply multiplying *t* by a scaling constant estimated by comparing the log-ratio thickness estimates with equivalent estimates derived from electron tomography. The error in the thickness estimate is given as the interquartile range of the thickness in the area of interest. Since the electron beam has a diameter of ∼1.5 µm, and the lamellae have a thickness gradient, the thickness will vary across the data-collection area.

### 2.10. Lamella thickness determination via cryo-ET

A cryo-ET tilt series on the lamella was acquired between ±60° relative to the lamella, with an increment of 3° using a dose symmetric data-acquisition scheme. A total dose of 100 e^−^ Å^−2^ was used with a dose per image of 2.44 e^−^ Å^−2^. Tilt series were collected with defocus settings between −3 and −6 µm, using the same imaging conditions as described above. The image data were saved in MRC format and the tomograms were reconstructed with the *ot2rec* automatic reconstruction pipeline (Perdigão *et al.*, 2022 ▸) using *IMOD* for the tomogram reconstruction (Mastronarde & Held, 2017 ▸). To extract a thickness measurement, slices through the centre of the reconstructed volume were taken and averaged over 10 voxels. The top and bottom layers of the lamellae were identified and the distance between them was estimated at the locations used for diffraction data collection.

## 3. Results and discussion

### 3.1. Datasets

Table 2 ▸ shows a summary of the datasets used in this analysis for each FIB source. Only datasets for which structure solution was successful were used in the analysis in the following sections: the table summarizes the reasons why some data were discarded. For some lamellae, there were errors during data collection. Of the datasets collected, some did not show any diffraction; this could be due to several reasons such as the sample was too thick, the beam was obscured at some tilt angles by ice contamination, the lamella was damaged or the data collection area was badly placed in a location outside the crystalline region of the lamella. Some datasets that displayed diffraction could not be processed successfully, typically because the spots could not be indexed, and others failed during structure solution. The number of datasets taken forward to the analysis is shown in the ‘Refinement successful’ row of Table 2 ▸. A dose series was collected for each lamella to validate the robustness and reproducibility of the experimental setup by measuring the effect of TEM beam damage on the sample, assuming the already known and well characterized behaviour of the diffraction in the presence of damage to the crystal (Storm *et al.*, 2020 ▸). For a dose series, the expected behaviour is for the strength and quality of the diffraction to drop as the crystal is damaged. Deviations from this behaviour could indicate issues with data collection or processing. In some cases, the data could not be processed for every dataset in the dose series; therefore, in the analysis of the data quality as a function of dose shown in Section S3 of the supporting information, only those lamellae for which all five datasets in the dose series could be processed were used. Likewise, only those lamellae which had an associated thickness measurement were used in the analysis of data quality as a function of lamella thickness. Sample selection criteria and success rates for 3DED of milled lamellae are rarely reported in the literature. We collected data from 118 lamellae across 16 grids. The percentage of lamellae which were successfully refined was 56% for argon, 80% for xenon and 77% for gallium. The lower success rate for argon resulted from a single grid where only 4 out of 14 lamellae produced successfully refined datasets with most datasets failing during indexing. This may reflect experimental error in this grid and excluding this grid would result in a higher success rate (77%). Across all grids, of the lamellae whose data were successfully refined and used in the analysis, 21% were observed to have some ice contamination visible in the diffraction patterns. In contrast, of the lamellae that were not used in the analysis for any of the reasons given above, 43% were observed to have some level of ice contamination. The median thickness of successful and unsuccessful datasets was 152 and 158 nm, respectively, across all grids.

### 3.2. Lamella thickness estimation

**Figure 3:**
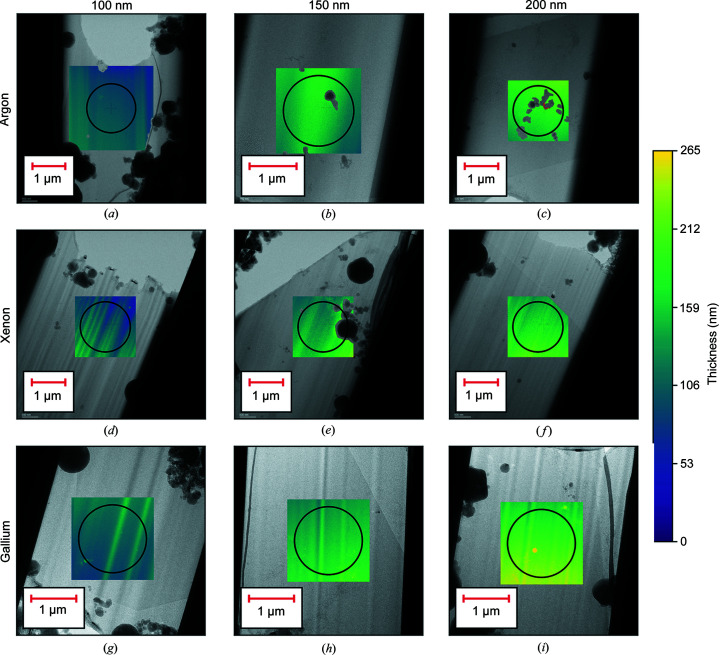
Thickness was estimated at the location of the data collection via the log-ratio method. The figures show the local thickness overlaid on an image of the lamella for lamellae milled with argon, xenon and gallium with approximate thicknesses of 100, 150 and 200 nm. The thickness of these lamellae ranges between ∼50 and ∼250 nm and is indicated by the colour bar which shows the thickness gradient over the lamellae. The area of the lamella under the selected area aperture is indicated by the black circle.

**Figure 4:**
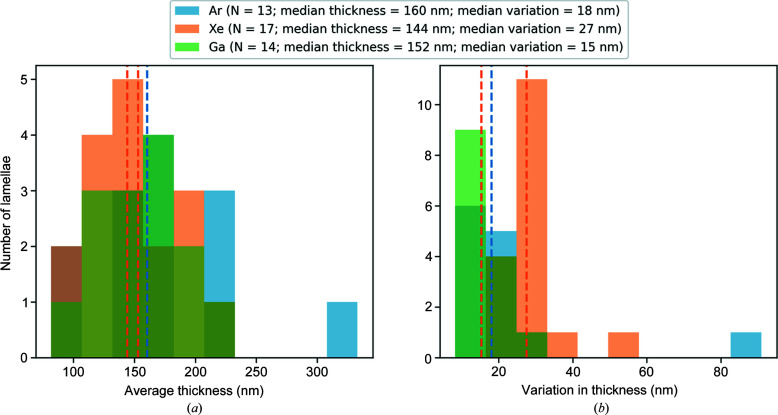
(*a*) Distribution of measured lamella thicknesses and (*b*) distribution of the variation in lamella thickness as measured by the interquartile range of the thickness within each lamella. The median thicknesses of a crystal milled with argon, xenon and gallium are 160, 144 and 152 nm, respectively. The median variation in thickness within a lamella milled with argon, xenon and gallium is 18, 27 and 15 nm, respectively.

The data collection positions were chosen to be as close as possible to the thin ends of the lamellae. Fig. 3 ▸ shows low-magnification images of a selection of lamellae with thickness maps covering the data collection area overlaid. Where a single thickness estimate for a lamella is given, this is the mean thickness within the data collection area at zero lamella tilt. The lamellae were milled with xenon and argon and have approximate thicknesses of 100, 150 and 200 nm. A gradient is visible in the heat maps representing the thickness in nanometres. The area covered by the selected area aperture is shown by the black circle. Fig. 4 ▸ shows the distribution of the lamella thicknesses as a function of milling plasma and the distribution of the variation in thickness across a lamella as measured by the interquartile range of thickness within the data collection area. Given the limited sample size, the distribution of lamella thicknesses is sparsely sampled. There was a substantial variation in the thickness of lamellae with the thinnest lamellae being around 90 nm and the thickest outliers being around 300 nm. On average, we were able to produce thinner lamellae using xenon than for argon and gallium with the median thicknesses being 160, 144 and 152 nm for crystals milled with argon, xenon, and gallium, respectively. The median variation in lamella thickness within the data collection area was 18 nm for argon, 27 nm for xenon and 15 nm for gallium. The larger variation in thickness for xenon may indicate a greater propensity for curtaining with this source. Note that, for a tilt angle of ±40°, which was the maximum tilt angle used in the 3DED data collection, the effective thickness of the lamellae will be ∼1.3× the thickness at zero tilt. Consequently, lamellae with zero tilt thicknesses of 100, 150 and 200 nm will have effective thicknesses of 130, 195 and 260 nm, respectively, at the maximum tilt angle.

### 3.3. Cryo-ET of crystal lamellae

**Figure 5:**
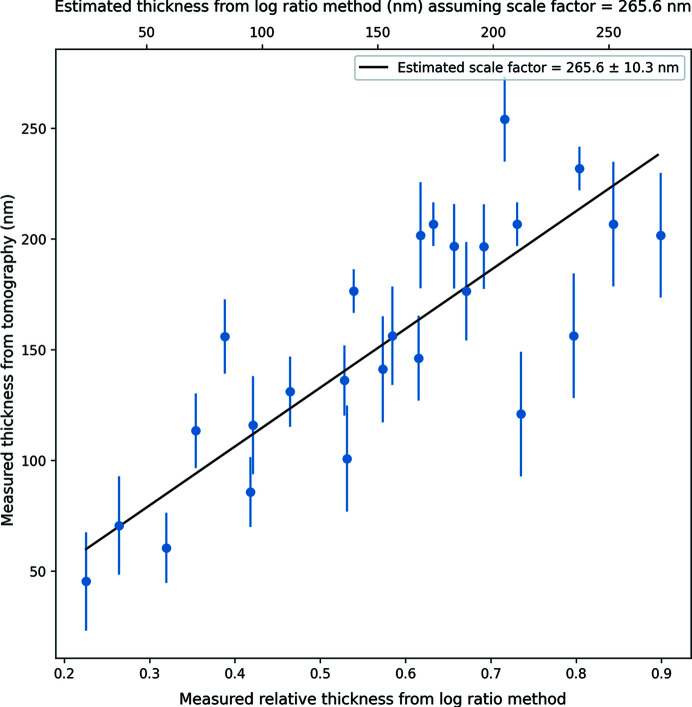
Thickness estimate obtained from tomography versus the thickness estimate obtained from the log-ratio method. The points represent multiple measurements from each lamella. The error bars indicate the standard error of the thicknesses recorded by the log-ratio and tomography methods, respectively. The black line indicates the fit to the data points with the slope giving an estimate of the constant scale factor to calibrate the log-ratio thickness measurements.

As previously mentioned, the purpose of collecting tomograms from the crystal lamellae was to provide an absolute measurement of the thickness to calibrate the log-ratio thickness measurements. There were multiple challenges associated with tomography of crystal lamellae. Automated tracking and focusing during data collection often failed because the periodic specimen was too uniform and, thus, the cross-correlation between images had multiple shallow minima. This occasionally resulted in movement of the area imaged, making reconstruction impossible. We were able to partly mitigate this problem by ensuring we imaged where there was nearby surface contamination which served as a unique feature for the tracking algorithms. The alignment of the individual tilt images suffered equally. Only when contamination was present in the area being imaged were we able to carry out the alignment. Additionally, once the images have been aligned, reconstruction may not work well for crystalline samples because diffraction effects weaken the assumptions used in standard back-projection algorithms. In the reconstructed volumes, it was also often difficult to visualize the top and bottom surfaces unless there was significant visible contamination. For some lamellae, only the top surface was visible. Despite these challenges we were able to visualize both surfaces for 8 lamellae and thus arrive at 26 independent measurements using both log-ratio and tomographic methods. These thickness estimates were compared, as shown in Fig. 5 ▸, and a straight-line fit to the data resulted in an estimate for the constant scale factor to apply to the log-ratio measurements of ∼265 ± 10.3 nm with a correlation of 0.81 and a root mean square error (RMSE) of 32 nm. The correlation between the targeted thickness at the milling stage and the measured thickness for the lamellae was poor with a correlation coefficient of 0.29. This suggests that the targeted thickness in the (p)FIB software is not a reliable estimate of the true sample thickness.

### 3.4. Electron diffraction data-processing statistics

**Figure 6:**
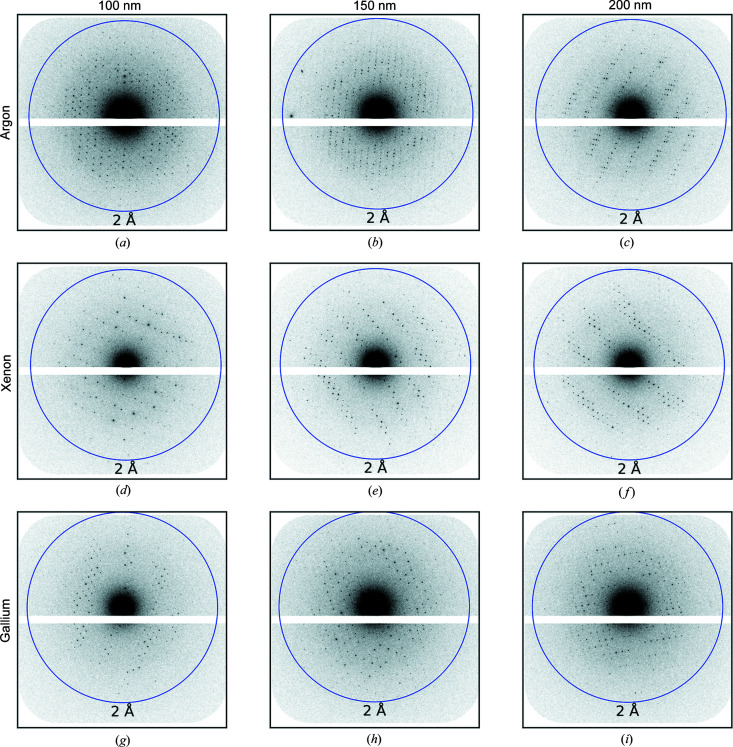
Diffraction patterns collected from the lamellae in thickness bins centred on thicknesses of (*a*), (*d*), (*g*) 100 nm; (*b*), (*e*), (*h*) 150 nm; and (*c*), (*f*), (*i*) 200 nm milled with (*a*)–(*c*) argon, (*d*)–(*f*) xenon and (*g*)–(*i*) gallium. The blue circles indicate a resolution of 2 Å.

Datasets from argon-, xenon- and gallium-milled crystals were independently binned into 50 nm intervals with thickness bins centred on 100, 150 and 200 nm. The number of datasets in the 100, 150 and 200 nm bins were (2, 6, 4) for argon, (5, 8, 4) for xenon and (3, 8, 3) for gallium, respectively. Fig. 6 ▸ shows a diffraction pattern, summed over ten frames, for a dataset in each thickness bin. The 2 Å-resolution ring is indicated by the blue circle in each case. For all datasets, the beam centre is clearly visible and the diffraction spots are sharp.

**Figure 7:**
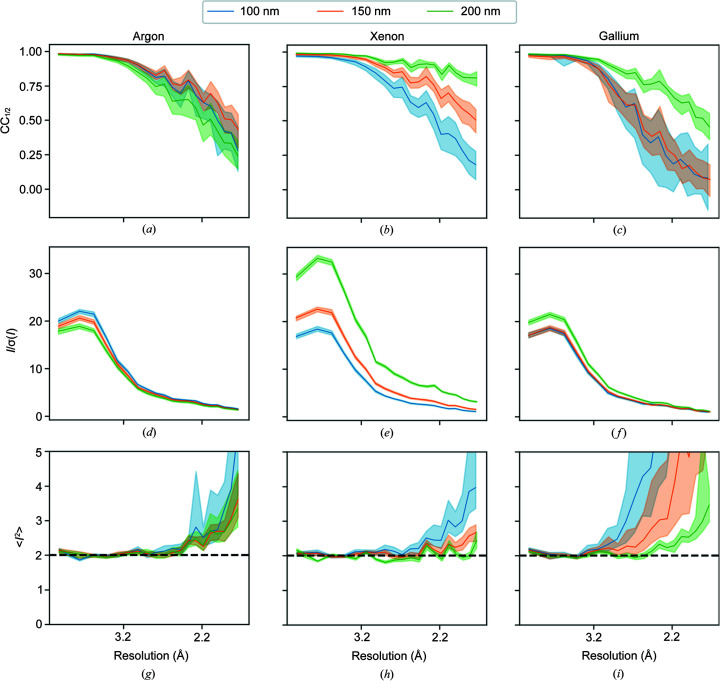
Data-processing statistics for lamellae milled with (*a*), (*d*), (*g*) argon; (*b*), (*e*), (*h*) xenon; and (*c*), (*f*), (*i*) gallium with thicknesses of approximately 100 (blue), 150 (orange) and 200 nm (green). The data quality is assessed by (*a*)–(*c*) CC_1/2_ curve versus resolution; (*d*)–(*f*) *I*/σ(*I*) versus resolution; and (*g*)–(*i*) the second moment of *I* versus resolution. The solid lines represent the median in each case and the shaded areas are the interquartile ranges. The dashed line in the second moment of the *I* plots shows the expected value for untwinned data (without considering noise). Tables S1, S2 and S3 in the supporting information show the range of data-processing statistics within the three thickness bins for all datasets for argon-, xenon- and gallium-milled crystals, respectively. Section S6 in the supporting information provides tables of data-processing statistics for all lamellae.

Fig. 7 ▸ shows the data-processing statistics for datasets within each thickness bin; the statistics for 100, 150 and 200 nm are shown in the same plots to aid comparison between lamellae of different thicknesses. The data from the xenon-milled crystals show more consistent behaviour than the data from the argon- and gallium-milled crystals. For xenon, the CC_1/2_ plots show that the data from thicker samples are of better quality than from thinner samples with the plots being clearly separated and showing progressively higher CC_1/2_ for 100, 150 and 200 nm samples. For gallium, there is no difference in data quality for 100 and 150 nm lamellae, except that the 100 nm samples show a greater variability. However, the 200 nm lamellae do show higher data quality. For the argon-milled crystals, there is essentially no difference in the CC_1/2_ plots for samples of different thicknesses.

The *I*/σ(*I*) plots in Figs. 7 ▸(*d*)–7 ▸(*f*) show that, for the xenon-milled lamellae, there is a clear difference in *I*/σ(*I*) for lamellae of different thicknesses. The thicker lamellae show higher *I*/σ(*I*), consistent with them having a larger volume of diffracting material. In contrast the variation in *I*/σ(*I*) between lamellae of different thicknesses for argon and gallium is small. For gallium, there is almost no difference for 100 and 150 nm samples with 200 nm samples having slightly higher *I*/σ(*I*). For argon, there is no significant difference in *I*/σ(*I*) for samples of different thicknesses. For 200 nm-thick samples, the xenon datasets typically have a higher *I*/σ(*I*) than the argon and gallium datasets.

Figs. 7 ▸(*g*)–7 ▸(*i*) shows the second moment of the intensities. For error-free data, the second acentric moment of the intensities at low resolution tends towards a value of 2 for untwinned data (Stein, 2007 ▸). When the variances in the intensities are considered, the moment tends to a value of 2 + var(*n*)/〈*I*〉^2^ (Parkhurst *et al.*, 2017 ▸) which increases towards infinity at high resolution. Deviations from the expected theoretical curve indicate bias in the reflection intensities. For very thick samples, this behaviour may change as a result of multiple scattering. The differences in the data quality between lamellae of different thicknesses become more apparent in the second moment plots. For each milling source, the second moments of the intensities from the 100 nm samples show greater variability than those from the 150 and 200 nm samples. Furthermore, the resolution at which the moments begin to increase towards infinity is lower for data from 100 nm samples, indicating that the data are more dominated by noise at higher resolution than the data from 150 and 200 nm samples. For argon, there is not much to distinguish the 150 and 200 nm data; however, for xenon and gallium, the plots for 150 and 200 nm data show progressively less variability and stay close to 2 until higher resolution, giving a clear indication that the data quality is better for the thicker samples.

### 3.5. Data quality as a function of milling plasma

**Figure 8:**
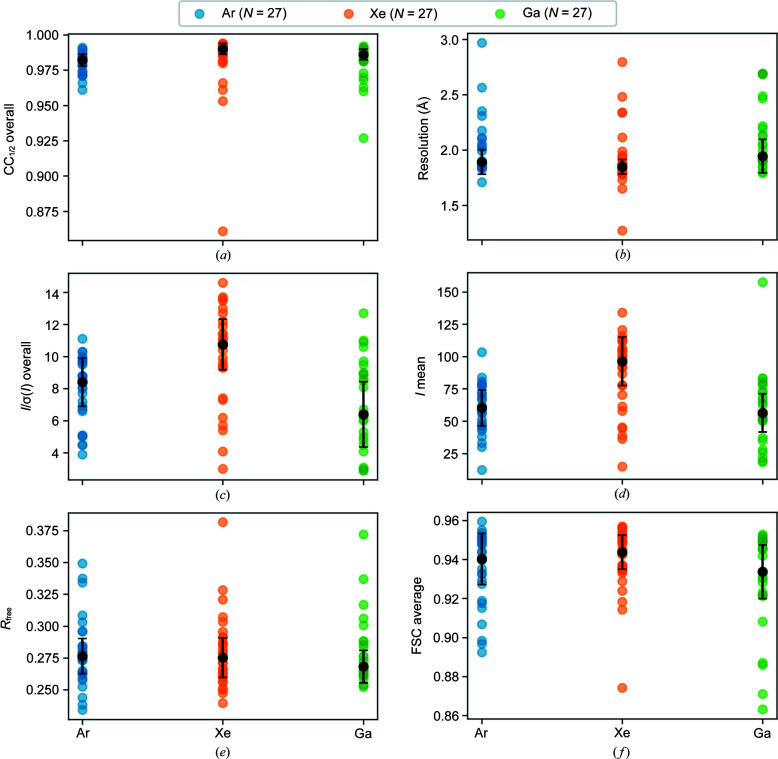
Data-processing statistics as a function of milling source: (*a*) CC_1/2_ overall to 2 Å; (*b*) resolution estimate based on CC_1/2_; (*c*) *I*/σ(*I*) overall to 2 Å; (*d*) *I* mean to 2 Å; (*e*) *R*_free_ to 2 Å; (*f*) FSC_average_ to 2 Å.

After obtaining an estimate of the resolution, each dataset was truncated to 2 Å to enable like-for-like comparison between the different datasets. This analysis compares the quality of diffraction data as a function of milling source using all datasets, which cover a range of measured thicknesses. As previously stated, the median thicknesses of the milled lamellae for argon-, xenon- and gallium-milled samples was 160, 144 and 152 nm, respectively. The quality of the diffraction data was evaluated using standard diffraction data-processing statistics: CC_1/2_, resolution estimate, *I*/σ(*I*), mean integrated intensity, *R*_free_ and FSC_average_ from the structure refinement. Analysis of variances (ANOVA) was used to test for statistical significance between the means of the data-processing statistics for each source. Fig. 8 ▸ shows the data quality as a function of milling source for argon, xenon and gallium. The spread of values for each source is shown along with the mean indicated by the black points and the interquartile range as indicated by the vertical error bars.

The overall CC_1/2_ to 2 Å is similar for each milling source: 0.99 ± 0.01 for xenon, 0.98 ± 0.01 for argon and 0.99 ± 0.01 for gallium. Likewise, the mean resolution was similar for each milling source: 1.85 ± 0.06 Å xenon, 1.89 ± 0.11 Å for argon and 1.94 ± 0.15 Å for gallium. Xenon-milled crystals display the highest average *I*/σ(*I*) and average *I* mean values. It is notable however that the xenon statistics typically show a greater variation around the mean. The *I*/σ(*I*) to 2 Å was 10.75 ± 1.57 for xenon, 8.40 ± 1.50 for argon and 6.4 ± 2.01 for gallium. The mean integrated intensity to 2 Å was 96.26 ± 18.75 for xenon, 60.26 ± 13.86 for argon and 56.37 ± 14.73 for gallium. After structure refinement, the model quality indicators were similar for each milling source. The *R*_free_ to 2 Å was 27.52 ± 1.55% for xenon, 27.64 ± 1.38% for argon and 26.82 ± 1.27 for gallium. The FSC_average_ to 2 Å was 0.94 ± 0.01 for xenon, 0.94 ± 0.01 for argon and 0.93 ± 0.01 for gallium. The difference in CC_1/2_, resolution, *R*_free_ and FSC_average_ between sources was not statistically significant (*p* = 0.95, *p* = 0.06, *p* = 0.99 and *p* = 0.18 respectively); however, the difference in *I*/σ(*I*) and mean integrated intensity was statistically significant (*p* < 0.01 and *p* < 0.01, respectively).

**Figure 9:**
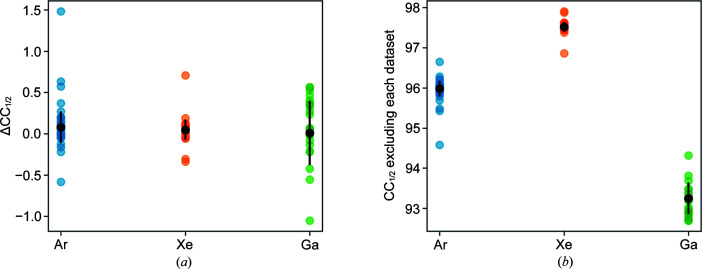
Combining multiple datasets for each milling source to assess cross-dataset consistency. (*a*) ΔCC_1/2_ to 2 Å; (*b*) CC_1/2_ to 2 Å excluding a single dataset at a time.

The milling rate for xenon has been shown to be at least twice that for argon and gallium (Brogden *et al.*, 2021 ▸; Berger *et al.*, 2022 ▸), so a possible explanation for the improved data quality with this plasma may be that reduced exposure to the milling source results in reduced damage. Fig. 9 ▸ shows the ΔCC_1/2_ analysis for combining multiple datasets for each plasma; the datasets collected from the xenon-milled crystals showed better consistency between datasets than the datasets collected from the argon- and gallium-milled crystals, with the gallium datasets showing significant variation.

**Figure 10:**
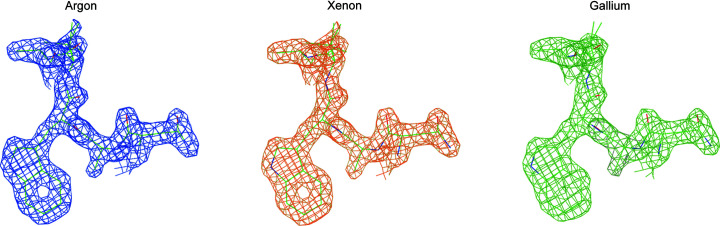
Atomic potential for a tryptophan with a contour level of 1.8σ for merged data from each milling source. Data were refined to the highest resolution achieved for each combined dataset, which was 1.75 Å for argon, 1.74 Å for xenon and 1.84 Å for gallium, respectively.

Fig. 10 ▸ shows atomic potential for a tryptophan W108 in lysozyme with a contour level of 1.8σ for merged data from each milling source to the highest resolution achieved for that dataset, which was 1.75 Å for argon, 1.74 Å for xenon and 1.84 Å for gallium, respectively. These maps indicate that the argon and xenon lamellae diffracted to higher resolution and the higher quality of the argon and xenon data is reflected in better visibility of the features in the ring. The merged data and refined models from each milling source are available to download from Zenodo (Parkhurst *et al.*, 2022 ▸).

### 3.6. Data quality as a function of lamella thickness

**Figure 11:**
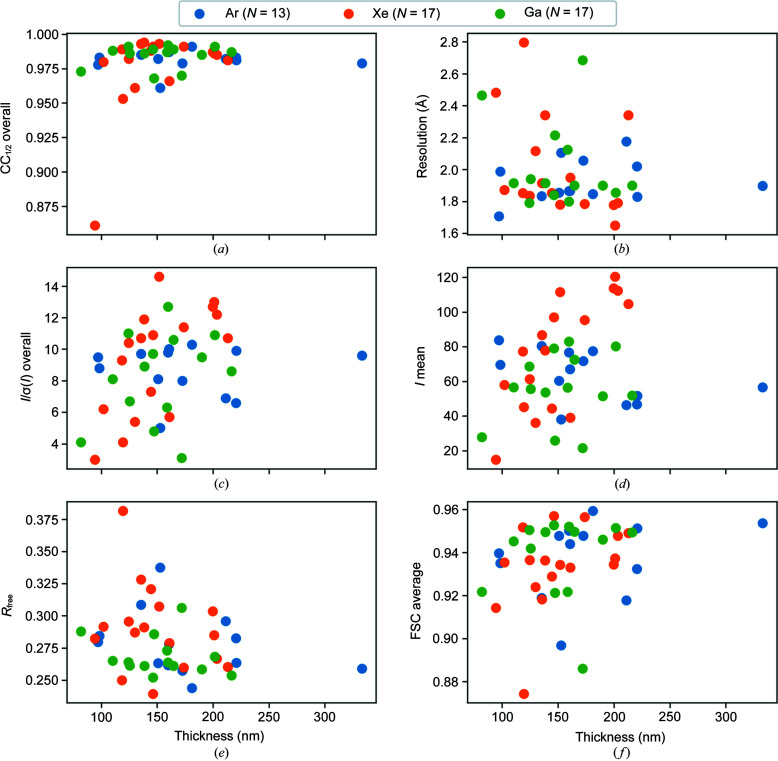
Data-processing statistics as a function of thickness: (*a*) CC_1/2_ overall to 2 Å; (*b*) resolution estimate based on CC_1/2_; (*c*) *I*/σ(*I*) overall to 2 Å; (*d*) *I* mean to 2 Å; (*e*) *R*_free_ to 2 Å; (*f*) FSC_average_ to 2 Å.

As shown in Fig. 11 ▸, the data quality was assessed as a function of lamella thickness from 85 to 300 nm. There is no strong trend in data quality with lamella thickness as measured by the overall CC_1/2_ to 2 Å. However, an upper bound on the thickness of the damage layer can be estimated. The thinnest lamellae, for which structure determination was successful, had thicknesses of ∼100 nm for argon, ∼90 nm for xenon and ∼85 nm for gallium, respectively. The lack of any thinner lamella measurements suggests limitations in producing lamellae that taper perfectly to zero at their ends. In this case it may be due to the fragility of protein crystals or reflect some mechanism of damage to the upper and lower lamellae surfaces that result in an imperfect edge. Assuming these measurements to be representative of the critical thickness would imply that the damage layer is at most 50, 45 and 42.5 nm on either side of the lamella for argon, xenon and gallium, respectively. Previously, based on experimental and theoretical considerations, it was estimated that, for lysozyme, ∼20 nm of undamaged crystal may be needed for structure solution from 3DED data; this would imply a damage layer of ∼40, ∼35 and ∼32.5 nm on each side of the lamella for argon, xenon and gallium, respectively.

The data quality indicators for the lamellae milled with argon and gallium appeared stable across the range of thicknesses considered, *i.e.* no trend was observed in the estimated resolution, *I*/σ(*I*) and mean integrated intensities as a function of thickness over this range. However, a trend can be seen for the xenon-milled lamellae which show an increase in the *I*/σ(*I*) and mean integrated intensity as a function of lamella thickness as well as an increase in estimated resolution. For the model quality indicators there appears to be a trend as a function of thickness. For the thinnest samples, *R*_free_ and FSC_average_ are significantly worse than at higher thickness, particularly for the xenon-milled crystals.

Out of the 44 lamellae with thickness estimates, only 4 were obtained with an average thickness in the data collection area of ≤100 nm. This is because it is technically challenging to avoid physical damage to the very thinnest lamella as described above. As previously shown in Fig. 3 ▸, this results in a distribution of lamella thicknesses that peaks around ∼150 nm for each milling source. A random sample of lamellae drawn from this distribution will tend to contain fewer lamellae with thicknesses below 100 nm than above 100 nm. Additionally, although the over-tilt method allows the far end of the lamella to be very thin, the electron beam has a finite size which necessarily covers a range of thicknesses due to the thickness gradient of the lamella. Therefore, the ability to reduce the average thickness within the data collection area is additionally limited by the geometry of the crystal and the beam size.

### 3.7. Simulation of the depth of ion implantation

**Figure 12:**
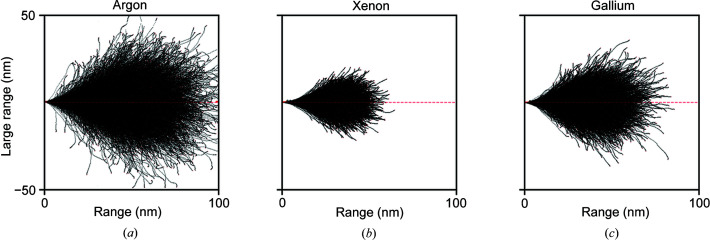
Ion trajectories from SRIM simulations for the transmission of (*a*) argon, (*b*) xenon and (*c*) gallium ions through lysozyme. The milling direction is assumed to be along the *x* axis. The lateral range (*y* axis) shows the distance the ion beam penetrates the sample orthogonal to the milling direction.

Monte Carlo simulations were performed using the *SRIM* software program (Ziegler *et al.*, 2010 ▸) in order to provide additional insight into the relative depths of the damage layer due to ion implantation for the xenon, argon and gallium sources at 30 keV. The density was assumed to be 1.35 g cm^−3^ (Egerton, 2015 ▸) and the relative number of atoms in the sample, taken from the PDB model (PDB entry 193L), was C (620), O (329), N (195), S (10), Cl (1), Na (1). In each case, the simulations were performed for 10 000 ion traces with an incidence angle of 0°. Note that the distributions of ion trajectories will differ depending on the incidence angle which, in turn, depend on the angle of the FIB beam with respect to the grid and the sample geometry. The results of the simulations can be seen in Fig. 12 ▸, which shows the distribution of ion trajectories for argon [Fig. 12 ▸(*a*)], xenon [Fig. 12 ▸(*b*)] and gallium [Fig. 12 ▸(*c*)]. The milling direction is assumed to be along the *x* axis. Therefore, the distance the ion beam penetrates the sample orthogonal to the milling direction is given by the lateral range on the *y* axis. The mean absolute value of the lateral range and the lateral straggle – defined as the second moment of the distribution – were (9.9, 12.6 nm) for argon, (4.3, 5.4 nm) for xenon and (6.5, 8.4 nm) for gallium. Although the mean absolute value of the lateral range is somewhat smaller than the depth of damage observed in practice, based on this, we would expect that the damage volume for xenon would be smaller than for argon and gallium, but the higher-energy density deposited in that smaller volume would result in greater localized damage. Modelling the absolute level of damage within the distribution of ion trajectories requires a model for damage per ion deposition and an accurate knowledge of the number of ions deposited per unit area. The true depth of observed damage will also be dependent on other factors such as the beam profile, focal stability and stage vibration as well as damage from secondary electrons and X-rays generated by the ion-sample interactions.

## 4. Conclusions

We have presented an analysis of the surface depth of damage caused by a plasma FIB during sample preparation by assessing the integrity of prepared lamellae. Sample integrity was quantified using ED data quality from lamellae of crystalline lysozyme as a pr­oxy. We analysed samples prepared using both argon and xenon plasmas and have compared the ED data quality of these lamellae with those prepared using a gallium ion source. The lamellae were milled with a shallow thickness gradient along the length of the lamella, resulting in a wedge lamella which facilitated the collection of diffraction datasets from various thicknesses ranging from 85 to 320 nm. The thicknesses of all lamellae were estimated using the log-ratio method and independently validated using cryo-ET. It is not possible using this data to determine where in the data collection area the diffraction is coming from so it is assumed to come from the whole data collection area and, as a result, we use the average thickness within that area. It would be interesting to determine how the curtaining might affect the diffraction on a finer level. This would require collecting diffraction data with a smaller electron beam size. However, such an experiment is beyond the scope of the current work.

Xenon-milling typically allowed the production of thinner lamellae and, on average, gave rise to higher-quality datasets, suggesting it is the least damaging. There is further subtle difference between argon and xenon. The xenon-milled data show a decrease in data quality with a decrease in thickness that would be consistent with a simple damage model. Argon data do not show this trend with data quality, rather it is relatively constant with decreasing thickness until a sharp cut-off in diffraction. We offer two explanations for the finding: that there is some artefact of the experiment that we have not identified, or that for argon there may be an additional damage mechanism at play. The data demonstrate that both plasma species are, at worst, no more damaging than gallium and suggest there may be a small improvement. We have observed that the use of microprobe illumination yields far superior diffraction images and data quality from protein crystal lamellae compared with the nanoprobe. This is likely due to the difference in how charge is accumulated and distributed on lamellae when using a microprobe and nanoprobe. However, further experiments are required to fully understand the issue and it is not yet clear whether collecting data with a larger beam size is intrinsically better or just easier from a user perspective.

Crystalline diffraction of proteins is a particularly sensitive tool for detecting damage. Taking the thinnest lamellae that resulted in usable diffraction data in our analysis as a guide to the extent of surface damage, the upper bound to the depth of the pFIB-milling damage layer is 50, 45 and 42.5 nm, for argon, xenon and gallium, respectively. This upper bound reflects both the potential for a damage layer of this thickness but also the practical limit in terms of the thinnest lamellae of crystals that can be routinely milled using the protocols described here. This upper bound is consistent with an analysis of the depth of milling damage from a gallium FIB using 2D template-matching which estimates that the FIB-milling damage may extend up to 60 nm from either surface of the lamella (Lucas & Grigorieff, 2023 ▸). We can estimate a lower bound for damage using an estimate of ∼20 nm of crystalline material (five unit cells) as the minimum for a high-quality crystallographic structure. This places the lower bound of damage layer thicknesses at 40, 35 and 32.5 nm for argon, xenon and gallium, respectively. This is consistent with a tomographic analysis of ribosome structure using the distance from the surface in argon-milled lamellae of HeLa cells which suggested a depth of damage of at least 30 nm (Berger *et al.*, 2022 ▸). With a damage layer of around 30 to 45 nm using pFIB, there may be issues of sample integrity in milling lamellae of 100 nm or below since the majority of the sample is influenced by the interaction with the plasma species. The utility of milling crystal lamellae for use in ED experiments is becoming apparent; however, progress is needed to make the technique more widely applicable. Currently, FIB-milling of crystals is a low-throughput technique; however, automation on next-generation FIB instruments should help to improve this. Additionally, more work is needed to optimize sample preparation conditions to enable a higher success rate for lamellae production. Finally, understanding and mitigating charging of lamellae in ED will enable advanced data-acquisition techniques that require the ability to use smaller electron beam sizes.

## Supplementary Material

Appendices S1 and S2 and Tables of statistics. DOI: 10.1107/S2052252523001902/ur5002sup1.pdf

## Figures and Tables

**Figure 13:**
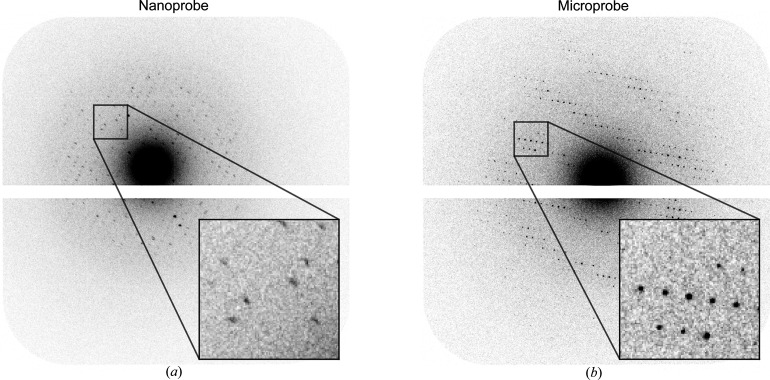
Examples of (*a*) ED data collection in nanoprobe mode where blurred diffraction spots are observed and (*b*) representative diffraction using microprobe mode. The white gap in the diffraction images is the physical gap between the panels of the detector. The diffraction in microprobe mode shows clear and well defined spots up to a resolution of ∼2 Å; however, the diffraction in nanoprobe mode shows blurry spots.

**Table 1:** Targeted probe currents used for the respective FIB sources and milling steps

Milling step	Spacing	Argon 30 kV	Xenon 30 kV	Gallium 30 kV
Coarse (1)	2 µm	2 nA	1 nA	0.3 nA
Coarse (2)	1 µm	0.74 nA	0.3 nA	0.1 nA
Polishing (3)	0.3 µm	60 pA	30 pA	30 pA

**Table 2:** The number of grids and lamellae used in the experiments and the progressive rejection of lamellae or data from the analysis at various stages of the experimental workflow due to problems described in the text ‘Refinement successful’ reflects the final number of datasets used in the full analysis with the percentages reflecting cumulative losses of lamellae from the analysis. The thickness measurement in the table refers to the number of lamellae with a thickness measurement from the log-ratio method as described in the text.

	Argon	Xenon	Gallium
No. of grids	6	4	6
No. of lamellae	48	35	35
Datasets measured	47 (98%)	34 (97%)	34 (97%)
Direct beam measured	46 (96%)	33 (94%)	33 (94%)
Diffraction observed	42 (88%)	31 (87%)	32 (91%)
Data reduction successful	28 (58%)	30 (86%)	27 (77%)
Refinement successful	27 (56%)	28 (80%)	27 (77%)
Complete dose series	18 (38%)	16 (46%)	19 (54%)
Thickness measured	13 (27%)	17 (49%)	14 (40%)

**Table 3:** Comparison of nanoprobe and microprobe data-collection modes quantifying the observation of datasets containing blurred diffraction spots

TEM mode	Source	No. of datasets	No. of blurry	Percentage blurry (%)
Nanoprobe	Argon	53	41	77
	Xenon	47	36	77
	Gallium	40	29	73
Microprobe	Argon	149	0	0
	Xenon	136	0	0
	Gallium	50	0	0

## Appendix A Microprobe versus nanoprobe

Initially we used a protocol for ED data collection using nanoprobe mode in the TEM (Beale *et al.*, 2020 ▸) but, in many cases, we observed blurry or misshapen diffuse spots as shown in Fig. 13 ▸(*a*). We manually inspected all the ED datasets we acquired from the lysozyme lamellae milled with argon, xenon and gallium to determine the fraction of datasets affected by this issue; these results are reported in Table 3 ▸ showing that, in each case, approximately 75% of datasets collected in nanoprobe mode exhibit blurred diffraction spots whereas microprobe mode with a selected area aperture resulted in no cases of blurred spots. We hypothesized this was the result of charging of the sample in the electron beam during data collection. It has previously been reported that the charging can be mitigated by applying a post-milling layer of sputtered platinum coating to the lamellae (Schaffer *et al.*, 2017 ▸; Beale *et al.*, 2020 ▸). The depth of the deposited platinum was hard to control precisely and introduced a complicating factor in the analysis since it effects contrast and surface chemistry. Application of platinum sputter coating did not appear to solve the problem of blurred diffraction spots. A method to correct for this effect during data collection is to interactively apply a small defocus to the images at the start of data collection. The rationale for doing this is that, if there is charging of the lamella in the electron beam, the lamella may act as a lens and deflect the beam. By changing the focus, the blurry spots can be made sharper on the detector plane, correcting for the defocussing caused by the lamella charging. However, this makes the data awkward to both collect and analyse, since a change in focus can cause a change in the position of the diffraction spots on the detector; therefore, the first few images affected by this procedure need to be discarded (Beale *et al.*, 2020 ▸).

We hypothesize that in nanoprobe mode, with a small beam size, the charge gradient is large enough to cause the lamella to act as an electron lens, defocusing the diffracted electrons. In microprobe mode, with a large beam size, the charge gradient is reduced, and the distortion of the spots is less apparent (Fig. 13 ▸). As microprobe mode irradiates, and therefore damages a much greater area of the sample, nanoprobe mode is usually preferred for ED in case other parts of a crystal need to be used for data collection. However, we clearly observed that the blurring problem was mitigated using microprobe mode with a selective area aperture in the TEM where we observed zero datasets with blurry spots. For all datasets reported here we therefore used microprobe mode and employed a selective area aperture to record only the diffraction from the desired area of the crystal. The blurring of spots has not been reported for ED collection using a thin needle or nano crystals, and we conclude that milling or lamella geometry may play a role in charge retention. A disadvantage to using microprobe mode with a large beam size is that, typically, the whole lamella is exposed, and therefore damaged, so multiple independent datasets cannot be acquired from distinct parts of the same lamella. Larger lamellae could enable this to some extent; however, larger lamellae may also have greater issues with mechanical stability.
