# Supplementary material for: Investigation of the milling characteristics of different focused-ion-beam sources assessed by three-dimensional electron diffraction from crystal lamellae
Source: IUCrJ. 2023 Mar 24;10(Pt 3):270–87. doi: 10.1107/S2052252523001902 (PMC10161776; doi:10.1107/S2052252523001902)
Supplement: Supplementary file 1 [file m-10-00270-sup1.pdf]

IUCrJ

Volume 10 (2023)

Supporting information for article:

Investigation of the milling characteristics of different focused-ion-beam sources assessed by three-dimensional electron diffraction from crystal lamellae

James M. Parkhurst, Adam D. Crawshaw, C. Alistair Siebert, Maud Dumoux, C. David Owen, Pedro Nunes, David Waterman, Thomas Glen, David I. Stuart, James H. Naismith and Gwyndaf Evans

S1. Data processing commands

The commands and parameters used to process the data are shown here:

```
# Import the data but manually specify some metadata
dials.import ${filename} \
  slow_fast_beam_centre=${beam_centre} \
  axis=0,-1,0 \
  geometry.scan.oscillation=0,${step_angle}\
  distance=${distance}

# Find the strong spots
dials.find_spots \
  imported.expt\
  filter.min_spot_size=3 \
  filter.d_min=3 \
  filter.d_max=10 \
  threshold.dispersion.gain=1.5 \
  threshold.dispersion.kernel_size=7,7

# Perform a few rounds of indexing and refinement
experiments=imported.expt
for i in $(seq 1 5)
do
  dials.index ${experiments} strong.refl \
    space_group=P43212 \
    detector.fix=all \
    beam.fix=all

  dials.refine indexed.{expt,refl} \
    scan_varying=False \
    unit_cell.restraints.tie_to_target.values=78.54,78.54,37.77,90,90,90 \
    unit_cell.restraints.tie_to_target.sigmas=0.05,0.05,0.05,0.05,0.05,0.05

  experiments=refined.expt
done

# Do the final scan varying geometry refinement
dials.refine refined.{expt,refl} \
  detector.fix=distance \
  scan_varying=True

# Integrate as normal
dials.integrate refined.{expt,refl}

# Check symmetry
dials.symmetry integrated.{expt,refl}

# Scale the data to a given resolution
dials.scale symmetrized.{expt,refl} d_min=${resolution}

# Merge the data
dials.merge scaled.{expt,refl}
```

S2. Structure solution and refinement commands

The commands and parameters used to do molecular replacement and refinement of the data are shown here:

```
# Convert cif to mtz
cif2mtz HKLIN 193l-sf.cif HKLOUT 193l.mtz

# Select the free set for validation from the reference reflections
freerflag \
  HKLIN 193l.mtz \
  HKLOUT 193l_free.mtz <<END
UNIQUE
FREEFRAC 0.05
END

# Set the free set for validation
cad hklin1 merged.mtz hklin2 193l_free.mtz hklout free.mtz
LABI FILE 1 ALLIn
LABI FILE 2 E1=FreeR_flag
END

# Randomise coordinates
pdbset \
  XYZIN 193l.pdb \
  XYZOUT 193l_randomised.pdb <<END
NOISE 0.4
END

# Do the molecular replacement
phaser <<END
TITLE lysozyme automatic
MODE MR_AUTO
HKLIN free.mtz
LABIN F=F SIGF=SIGF
ENSEMBLE 193l PDB 193l_randomised.pdb IDENTITY 100
SEARCH ENSEMBLE 193l NUM 1
ROOT phased
END

# Do the refinement
refmac5 \
  HKLIN free.mtz \
  HKLOUT refined.mtz \
  XYZIN phased.1.pdb \
  MAPOUT refined.map \
  XYZOUT refined.pdb <<END
SOURCE EM MB
NCYCLES 80
WEIGHT AUTO
MAKE HYDR ALL
MAKE HOUT YES
REFI BREF ISOT
MAKE NEWLIGAND EXIT
SCALE TYPE SIMPLE
SOLVENT YES
PHOUT
MONI DIST 1000000
PDBOUT KEEP TRUE
END
```

S3. Data quality as a function of electron dose

For each lamella, five ED datasets were collected at the same data collection position and the data quality was evaluated as a function of electron dose as shown in Figure S1 which shows the overall $CC_{1/2}$ to 2 Å, estimated resolution, overall I/sig(I) to 2 Å and mean integrated intensity to 2 Å. In each plot, the point is the median of all datasets, and the error bars show the interquartile range. For a dose series, the expected behaviour is for the strength and quality of the diffraction to drop as the crystal is damaged. Deviations from this behaviour could indicate issues with data collection or processing. The analysis demonstrates that the data were well collected and integrated. As expected, there is a trend of decrease in I/sig(I) and mean integrated intensity with increasing electron dose indicative of damage from the electron beam. In each case, the xenon milled crystals have higher mean integrated intensity and I / sig(I) than the argon and gallium milled crystals. Although the gallium milled crystals exhibit higher mean integrated intensity than argon milled crystals as a function of dose, they show a lower I / sig(I). The xenon milled crystals also show a better overall $CC_{1/2}$ and better estimated resolution than the argon and gallium milled crystals. Although, the argon milled crystals show lower overall $CC_{1/2}$ than the gallium milled crystals, they, nevertheless, have a higher estimated resolution. This trend is also carried over into the refinement statistics where the xenon milled crystals have consistently better R_{free} and $FSC_{average}$ to 2 Å than the other sources, followed by argon milled crystals and then gallium milled crystals. It should be noted that for the analysis as a function of dose, datasets were only included if all 5 datasets in the dose series were able to be processed. This resulted in an exclusion of some poorer quality datasets. The fraction of datasets used in the analysis which had complete dose series was 18 / 27 for argon, 16 / 28 for xenon, and 19 / 27 for gallium.

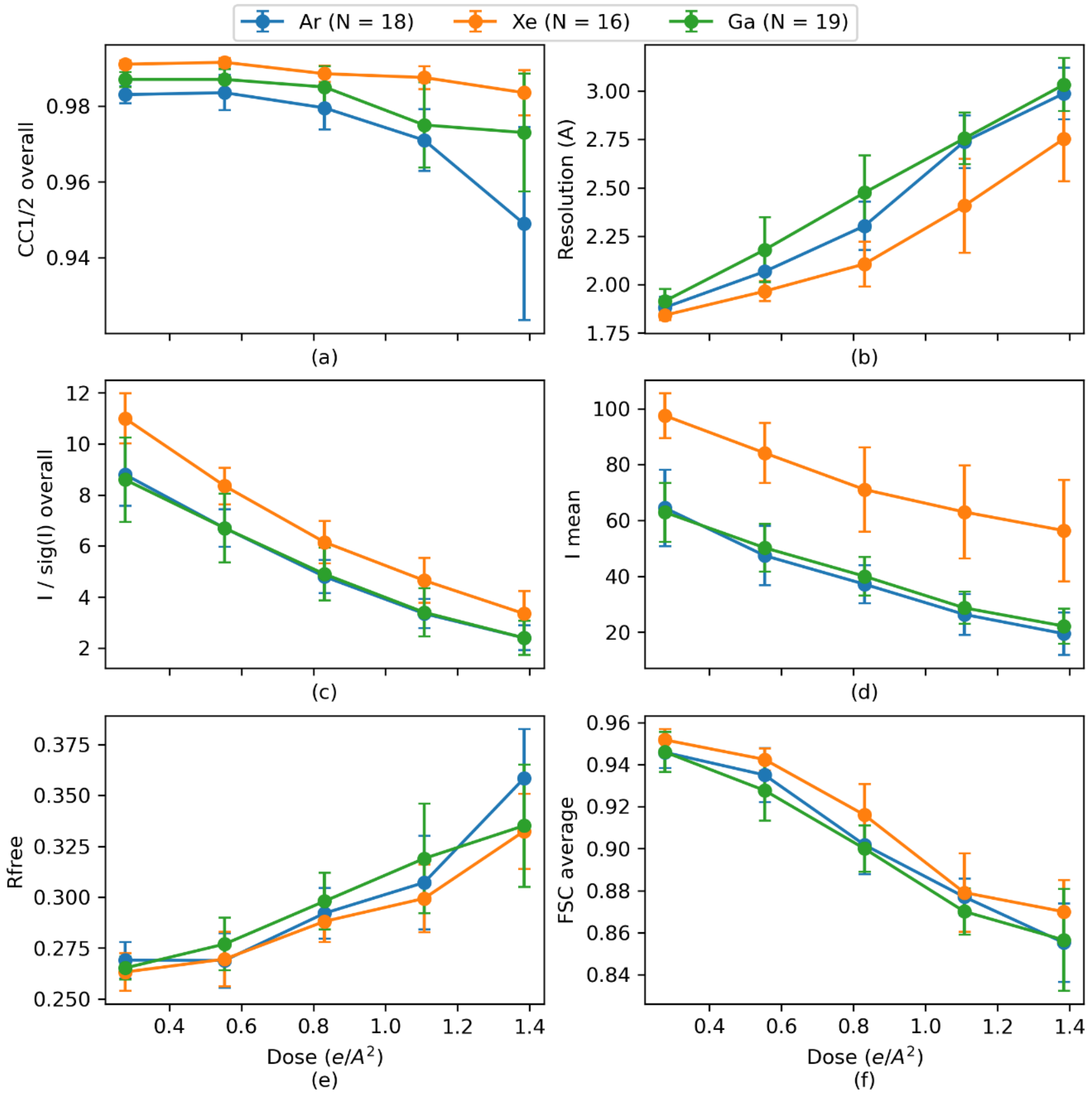

Figure S1 Data processing statistics as a function of dose: (a) CC1/2 overall to 2 Å; (b) resolution estimate based on CC1/2; (c) I/sig(I) overall to 2 Å; (d) I mean to 2 Å; (e) Rfree to 2 Å; (f) FSCaverage to 2 Å.

S4. Electron diffraction data processing statistics summary

Tables S1, S2 and S3 show the range of the overall summary statistics for datasets binned by lamella thickness for lamellae milled with argon, xenon and gallium respectively.

Table S1 Data processing statistics for lamellae of different thicknesses (100 nm, 150 nm, and 200 nm) milled with argon. The values given are the range from all datasets within in each thickness bin.

	100 nm	150 nm	200 nm
Num datasets	2	6	4
Resolution (Å)	1.71 - 1.99	1.83 - 2.10	1.83 - 2.17
Multiplicity	4.40 - 8.20	3.40 - 7.20	5.70 - 8.20
Completeness	45.71 - 69.15	48.76 - 90.73	73.27 - 96.85
Mean I/σ(I)	5.20 - 5.60	4.60 - 5.60	4.90 - 6.60
Rmerge	0.24 - 0.34	0.25 - 0.34	0.24 - 0.44
Rmeas	0.28 - 0.37	0.29 - 0.37	0.27 - 0.47
Rpim	0.12 - 0.13	0.11 - 0.15	0.11 - 0.16
CC½	0.98 - 0.98	0.95 - 0.98	0.97 - 0.99
R free	0.28 - 0.28	0.26 - 0.34	0.24 - 0.30
FSC average	0.94 - 0.94	0.90 - 0.95	0.92 - 0.96

Table S2 Data processing statistics for lamellae of different thicknesses (100 nm, 150 nm, and 200 nm) milled with xenon. The values given are the range from all datasets within in each thickness bin.

	100 nm	150 nm	200 nm
Num datasets	5	8	4
Resolution (Å)	1.84 - 2.80	1.78 - 2.34	1.65 - 2.34
Multiplicity	2.70 - 6.30	3.70 - 6.50	4.60 - 5.00
Completeness	44.03 - 88.54	42.56 - 100.00	46.87 - 59.56
Mean I/σ(I)	3.00 - 10.40	5.40 - 14.60	10.70 - 13.00
Rmerge	0.17 - 0.51	0.15 - 0.43	0.16 - 0.17
Rmeas	0.19 - 0.59	0.17 - 0.48	0.18 - 0.20
Rpim	0.08 - 0.31	0.07 - 0.22	0.08 - 0.09
CC½	0.86 - 0.99	0.96 - 0.99	0.98 - 0.99
R free	0.25 - 0.38	0.24 - 0.33	0.26 - 0.30
FSC average	0.87 - 0.95	0.92 - 0.96	0.93 - 0.95

Table S3 Data processing statistics for lamellae of different thicknesses (100 nm, 150 nm, and 200 nm) milled with gallium. The values given are the range from all datasets within in each thickness bin.

	100 nm	150 nm	200 nm
Num datasets	3	8	3
Resolution (Å)	1.79 - 2.46	1.80 - 2.69	1.85 - 1.90
Multiplicity	5.90 - 6.60	4.40 - 7.70	5.70 - 7.20
Completeness	79.36 - 91.96	77.14 - 97.83	72.78 - 94.44
Mean I/σ(I)	4.10 - 11.00	3.10 - 12.70	8.60 - 10.90
Rmerge	0.18 - 0.37	0.17 - 0.43	0.19 - 0.21
Rmeas	0.20 - 0.40	0.19 - 0.48	0.20 - 0.23
Rpim	0.07 - 0.17	0.07 - 0.20	0.07 - 0.10
CC½	0.97 - 0.99	0.97 - 0.99	0.98 - 0.99
R free	0.26 - 0.29	0.25 - 0.31	0.25 - 0.27
FSC average	0.92 - 0.95	0.89 - 0.95	0.95 - 0.95

S5. Electron diffraction data processing statistics merged by milling source

Tables S4, S5 and S6 contain summary statistics for the data merged from all lamellae milled with argon xenon and gallium respectively. These data were used to produce the maps in Figure 10 in the main text.

Table S4 Summary statistics for the data merged from all argon milled lamellae.

	Overall	Low resolution	High resolution
Resolution (Å)	78.60 - 1.70	78.68 - 4.61	1.73 - 1.70
Multiplicity	133.0	110.5	84.2
Completeness	100.00%	100.00%	100.00%
Mean I/σ(I)	24.8	98.4	0.9
Rmerge	0.570	0.180	6.930
Rmeas	0.572	0.181	6.970
Rpim	0.047	0.016	0.707
CC½	0.997	0.993	0.070
Rfree	0.242		
FSC average	0.941		

Table S5 Summary statistics for the data merged from all xenon milled lamellae.

	Overall	Low resolution	High resolution
Resolution (Å)	78.55 - 1.70	78.63 - 4.61	1.73 - 1.70
Multiplicity	122.6	101.8	78.0
Completeness	100.00%	100.00%	100.00%
Mean I/σ(I)	30.2	116.9	1.0
Rmerge	0.301	0.131	7.057
Rmeas	0.302	0.132	7.106
Rpim	0.027	0.013	0.776
CC½	0.999	0.999	0.078
Rfree	0.241		
FSC average	0.952		

Table S6 Summary statistics for the data merged from all gallium milled lamellae.

	Overall	Low resolution	High resolution
Resolution (Å)	55.60 - 1.70	55.63 - 4.61	1.73 - 1.70
Multiplicity	147.3	128.4	91.6
Completeness	97.89%	97.78%	96.74%
Mean I/σ(I)	23.3	104.9	0.6
Rmerge	0.559	0.160	12.786
Rmeas	0.561	0.161	12.873
Rpim	0.047	0.014	1.383
CC½	0.998	0.999	0.044
Rfree	0.244		
FSC average	0.941		

S6. Electron diffraction data processing statistics per dataset

Table S7 Data processing statistics for all the individually processed ED datasets.

xenon: grid 1; lamella 1			
	Overall	Low resolution	High resolution
Resolution (Å)	55.65 - 2.00	55.67 - 5.43	2.03 - 2.00
Multiplicity	5.2	4.0	5.4
Completeness	81.05%	79.92%	80.15%
Mean I/σ(I)	7.4	21.9	1.1
Rmerge	0.196	0.097	1.024
Rmeas	0.218	0.112	1.140
Rpim	0.093	0.053	0.484
CC½	0.989	0.992	0.512
Rfree	0.286		
FSC average	0.936		
Thickness	-		

xenon: grid 1; lamella 2			
	Overall	Low resolution	High resolution
Resolution (Å)	55.07 - 2.00	55.09 - 5.43	2.03 - 2.00
Multiplicity	5.2	4.2	5.3
Completeness	88.54%	86.29%	88.89%
Mean I/σ(I)	9.3	27.0	1.3
Rmerge	0.174	0.086	0.858
Rmeas	0.194	0.099	0.957
Rpim	0.082	0.047	0.409
CC½	0.989	0.990	0.628
Rfree	0.250		
FSC average	0.952		
Thickness	118		

xenon: grid 1; lamella 3			
	Overall	Low resolution	High resolution
Resolution (Å)	55.55 - 2.00	55.57 - 5.43	2.03 - 2.00
Multiplicity	3.7	2.9	3.9
Completeness	98.68%	97.54%	98.78%
Mean I/σ(I)	5.4	14.9	1.1
Rmerge	0.426	0.188	1.930
Rmeas	0.481	0.224	2.156
Rpim	0.218	0.117	0.937
CC½	0.961	0.978	0.298
Rfree	0.287		
FSC average	0.924		
Thickness	129		

xenon: grid 1; lamella 4			
	Overall	Low resolution	High resolution
Resolution (Å)	78.32 - 2.00	78.38 - 5.43	2.03 - 2.00
Multiplicity	5.6	4.2	5.8
Completeness	100.00%	100.00%	100.00%
Mean I/σ(I)	10.9	28.3	2.1
Rmerge	0.171	0.094	0.594
Rmeas	0.189	0.107	0.651
Rpim	0.078	0.050	0.260
CC½	0.991	0.992	0.779
Rfree	0.239		
FSC average	0.957		
Thickness	146		

xenon: grid 1; lamella 6			
	Overall	Low resolution	High resolution
Resolution (Å)	55.46 - 2.00	55.48 - 5.43	2.03 - 2.00
Multiplicity	6.3	5.2	6.5
Completeness	79.29%	75.93%	80.84%
Mean I/σ(I)	6.2	19.7	0.8
Rmerge	0.276	0.117	1.392
Rmeas	0.302	0.131	1.514
Rpim	0.118	0.057	0.584
CC½	0.980	0.987	0.497
Rfree	0.292		
FSC average	0.935		
Thickness	101		

xenon: grid 1; lamella 7			
	Overall	Low resolution	High resolution
Resolution (Å)	55.07 - 2.00	55.09 - 5.43	2.03 - 2.00
Multiplicity	5.8	4.5	5.9
Completeness	55.81%	55.31%	57.01%
Mean I/σ(I)	11.9	30.7	1.8
Rmerge	0.154	0.078	0.697
Rmeas	0.169	0.089	0.763
Rpim	0.068	0.041	0.303
CC½	0.994	0.996	0.833
Rfree	0.291		
FSC average	0.936		
Thickness	138		

xenon: grid 1; lamella 8			
	Overall	Low resolution	High resolution
Resolution (Å)	55.44 - 2.00	55.46 - 5.43	2.03 - 2.00
Multiplicity	6.1	4.8	6.3
Completeness	83.63%	81.04%	84.17%
Mean I/σ(I)	11.4	30.0	2.3
Rmerge	0.173	0.094	0.604
Rmeas	0.189	0.107	0.658
Rpim	0.075	0.048	0.257
CC½	0.991	0.989	0.834
Rfree	0.260		
FSC average	0.957		
Thickness	173		

xenon: grid 1; lamella 9			
	Overall	Low resolution	High resolution
Resolution (Å)	55.49 - 2.00	55.51 - 5.43	2.03 - 2.00
Multiplicity	6.5	5.3	6.7
Completeness	87.46%	83.09%	87.78%
Mean I/σ(I)	7.3	21.1	1.3
Rmerge	0.254	0.130	1.071
Rmeas	0.276	0.144	1.160
Rpim	0.106	0.061	0.438
CC½	0.988	0.988	0.665
Rfree	0.321		
FSC average	0.929		
Thickness	144		

argon: grid 2; lamella 1			
	Overall	Low resolution	High resolution
Resolution (Å)	55.47 - 2.00	55.49 - 5.43	2.03 - 2.00
Multiplicity	4.5	3.6	4.6
Completeness	76.25%	75.10%	77.97%
Mean I/σ(I)	5.1	16.5	0.7
Rmerge	0.291	0.133	1.600
Rmeas	0.331	0.156	1.821
Rpim	0.151	0.078	0.833
CC½	0.974	0.978	0.723
Rfree	0.334		
FSC average	0.915		
Thickness	-		

argon: grid 2; lamella 4			
	Overall	Low resolution	High resolution
Resolution (Å)	55.53 - 2.00	55.55 - 5.43	2.03 - 2.00
Multiplicity	5.0	3.8	5.3
Completeness	31.04%	32.43%	31.20%
Mean I/σ(I)	4.5	13.1	0.8
Rmerge	0.323	0.141	1.311
Rmeas	0.360	0.163	1.452
Rpim	0.155	0.081	0.609
CC½	0.966	0.985	0.347
Rfree	0.349		
FSC average	0.892		
Thickness	-		

argon: grid 2; lamella 9			
	Overall	Low resolution	High resolution
Resolution (Å)	55.83 - 2.00	55.86 - 5.43	2.03 - 2.00
Multiplicity	4.4	3.5	4.5
Completeness	71.21%	70.06%	70.59%
Mean I/σ(I)	8.8	22.8	1.9
Rmerge	0.196	0.100	0.641
Rmeas	0.222	0.118	0.720
Rpim	0.099	0.060	0.315
CC½	0.984	0.990	0.674
Rfree	0.273		
FSC average	0.940		
Thickness	-		

argon: grid 2; lamella 13			
	Overall	Low resolution	High resolution
Resolution (Å)	55.74 - 2.00	55.77 - 5.43	2.03 - 2.00
Multiplicity	3.4	2.7	3.5
Completeness	92.09%	90.70%	92.25%
Mean I/σ(I)	8.1	20.6	1.7
Rmerge	0.181	0.095	0.658
Rmeas	0.213	0.119	0.768
Rpim	0.110	0.070	0.386
CC½	0.982	0.988	0.717
Rfree	0.263		
FSC average	0.948		
Thickness	150		

argon: grid 3; lamella 7			
	Overall	Low resolution	High resolution
Resolution (Å)	37.69 - 2.00	37.70 - 5.42	2.03 - 2.00
Multiplicity	7.4	5.9	7.5
Completeness	64.82%	61.32%	65.59%
Mean I/σ(I)	10.0	26.1	1.8
Rmerge	0.229	0.132	0.859
Rmeas	0.246	0.146	0.919
Rpim	0.087	0.059	0.313
CC½	0.987	0.987	0.713
Rfree	0.278		
FSC average	0.944		
Thickness	160		

argon: grid 3; lamella 8			
	Overall	Low resolution	High resolution
Resolution (Å)	78.46 - 2.00	78.52 - 5.43	2.03 - 2.00
Multiplicity	5.2	4.1	5.3
Completeness	97.33%	97.12%	97.56%
Mean I/σ(I)	7.2	20.6	1.1
Rmerge	0.222	0.110	1.117
Rmeas	0.248	0.126	1.246
Rpim	0.105	0.060	0.534
CC½	0.982	0.978	0.489
Rfree	0.234		
FSC average	0.952		
Thickness	-		

argon: grid 3; lamella 9			
	Overall	Low resolution	High resolution
Resolution (Å)	55.54 - 2.00	55.56 - 5.43	2.03 - 2.00
Multiplicity	5.9	4.6	6.0
Completeness	80.40%	79.06%	80.49%
Mean I/σ(I)	9.8	25.3	1.9
Rmerge	0.182	0.107	0.649
Rmeas	0.200	0.122	0.712
Rpim	0.080	0.056	0.280
CC½	0.987	0.988	0.723
Rfree	0.262		
FSC average	0.950		
Thickness	159		

argon: grid 3; lamella 10			
	Overall	Low resolution	High resolution
Resolution (Å)	55.78 - 2.00	55.80 - 5.43	2.03 - 2.00
Multiplicity	5.4	4.2	5.6
Completeness	81.44%	80.29%	80.59%
Mean I/σ(I)	6.6	18.9	1.2
Rmerge	0.236	0.119	1.017
Rmeas	0.263	0.137	1.123
Rpim	0.111	0.065	0.463
CC½	0.983	0.986	0.545
Rfree	0.283		
FSC average	0.932		
Thickness	220		

argon: grid 3; lamella 11			
	Overall	Low resolution	High resolution
Resolution (Å)	55.35 - 2.00	55.37 - 5.43	2.03 - 2.00
Multiplicity	5.6	4.4	5.8
Completeness	84.39%	82.14%	85.19%
Mean I/σ(I)	10.3	27.7	1.8
Rmerge	0.178	0.092	0.731
Rmeas	0.196	0.105	0.800
Rpim	0.081	0.049	0.320
CC½	0.991	0.993	0.787
Rfree	0.244		
FSC average	0.959		
Thickness	181		

argon: grid 3; lamella 12			
	Overall	Low resolution	High resolution
Resolution (Å)	55.52 - 2.00	55.54 - 5.43	2.03 - 2.00
Multiplicity	6.6	5.2	6.6
Completeness	76.81%	75.00%	77.59%
Mean I/σ(I)	8.6	22.9	1.6
Rmerge	0.271	0.155	1.030
Rmeas	0.294	0.174	1.107
Rpim	0.108	0.075	0.385
CC½	0.971	0.954	0.649
Rfree	0.279		
FSC average	0.944		
Thickness	-		

argon: grid 3; lamella 13			
	Overall	Low resolution	High resolution
Resolution (Å)	55.55 - 2.00	55.57 - 5.43	2.03 - 2.00
Multiplicity	5.4	4.1	5.6
Completeness	85.74%	84.02%	85.54%
Mean I/σ(I)	9.6	24.2	2.1
Rmerge	0.214	0.120	0.693
Rmeas	0.238	0.139	0.766
Rpim	0.101	0.068	0.316
CC½	0.979	0.975	0.618
Rfree	0.259		
FSC average	0.954		
Thickness	333		

xenon: grid 4; lamella 4			
	Overall	Low resolution	High resolution
Resolution (Å)	55.46 - 2.00	55.48 - 5.43	2.03 - 2.00
Multiplicity	4.8	3.6	4.8
Completeness	46.87%	47.30%	46.94%
Mean I/σ(I)	12.7	28.3	3.4
Rmerge	0.164	0.094	0.427
Rmeas	0.185	0.113	0.476
Rpim	0.083	0.060	0.206
CC½	0.987	0.989	0.809
Rfree	0.303		
FSC average	0.934		
Thickness	199		

xenon: grid 4; lamella 5			
	Overall	Low resolution	High resolution
Resolution (Å)	55.34 - 2.00	55.36 - 5.43	2.03 - 2.00
Multiplicity	4.7	3.6	5.0
Completeness	58.80%	56.85%	59.85%
Mean I/σ(I)	12.2	29.5	2.9
Rmerge	0.172	0.094	0.510
Rmeas	0.195	0.113	0.568
Rpim	0.088	0.060	0.246
CC½	0.985	0.987	0.822
Rfree	0.267		
FSC average	0.948		
Thickness	203		

xenon: grid 4; lamella 6			
	Overall	Low resolution	High resolution
Resolution (Å)	55.70 - 2.00	55.72 - 5.43	2.03 - 2.00
Multiplicity	5.3	4.1	5.5
Completeness	42.56%	42.65%	42.36%
Mean I/σ(I)	10.7	26.4	2.3
Rmerge	0.171	0.084	0.621
Rmeas	0.189	0.096	0.683
Rpim	0.079	0.045	0.280
CC½	0.993	0.997	0.757
Rfree	0.328		
FSC average	0.918		
Thickness	135		

xenon: grid 4; lamella 7			
	Overall	Low resolution	High resolution
Resolution (Å)	37.26 - 2.00	37.27 - 5.42	2.03 - 2.00
Multiplicity	4.1	3.2	4.3
Completeness	71.54%	69.52%	72.07%
Mean I/σ(I)	5.7	14.3	1.2
Rmerge	0.257	0.130	0.948
Rmeas	0.296	0.153	1.079
Rpim	0.135	0.077	0.482
CC½	0.966	0.984	0.363
Rfree	0.279		
FSC average	0.933		
Thickness	160		

xenon: grid 4; lamella 8			
	Overall	Low resolution	High resolution
Resolution (Å)	55.55 - 2.00	55.57 - 5.43	2.03 - 2.00
Multiplicity	5.7	4.3	6.0
Completeness	45.76%	46.07%	45.52%
Mean I/σ(I)	10.4	24.5	2.8
Rmerge	0.229	0.128	0.595
Rmeas	0.252	0.146	0.651
Rpim	0.101	0.068	0.251
CC½	0.982	0.981	0.659
Rfree	0.296		
FSC average	0.936		
Thickness	124		

xenon: grid 5; lamella 1			
	Overall	Low resolution	High resolution
Resolution (Å)	56.11 - 2.00	56.13 - 5.43	2.03 - 2.00
Multiplicity	3.5	2.8	3.5
Completeness	59.31%	57.53%	58.23%
Mean I/σ(I)	4.1	13.8	0.6
Rmerge	0.505	0.156	2.883
Rmeas	0.589	0.190	3.297
Rpim	0.295	0.106	1.566
CC½	0.953	0.985	0.132
Rfree	0.382		
FSC average	0.874		
Thickness	119		

xenon: grid 5; lamella 2			
	Overall	Low resolution	High resolution
Resolution (Å)	56.19 - 2.00	56.22 - 5.43	2.03 - 2.00
Multiplicity	5.8	4.2	5.9
Completeness	46.09%	47.30%	46.63%
Mean I/σ(I)	14.6	32.6	3.7
Rmerge	0.153	0.084	0.464
Rmeas	0.168	0.096	0.506
Rpim	0.068	0.045	0.199
CC½	0.993	0.994	0.838
Rfree	0.307		
FSC average	0.934		
Thickness	151		

xenon: grid 5; lamella 3			
	Overall	Low resolution	High resolution
Resolution (Å)	55.32 - 2.00	55.35 - 5.43	2.03 - 2.00
Multiplicity	5.0	3.7	5.0
Completeness	50.15%	49.79%	49.13%
Mean I/σ(I)	13.0	30.4	3.2
Rmerge	0.165	0.096	0.448
Rmeas	0.186	0.113	0.502
Rpim	0.083	0.059	0.219
CC½	0.986	0.982	0.782
Rfree	0.285		
FSC average	0.937		
Thickness	201		

xenon: grid 5; lamella 5			
	Overall	Low resolution	High resolution
Resolution (Å)	37.50 - 2.00	37.50 - 5.42	2.03 - 2.00
Multiplicity	2.7	2.1	2.7
Completeness	44.03%	42.03%	44.63%
Mean I/σ(I)	3.0	7.2	0.6
Rmerge	0.443	0.203	1.559
Rmeas	0.546	0.261	1.895
Rpim	0.306	0.159	1.035
CC½	0.861	0.937	0.083
Rfree	0.282		
FSC average	0.914		
Thickness	94		

xenon: grid 5; lamella 6			
	Overall	Low resolution	High resolution
Resolution (Å)	55.47 - 2.00	55.50 - 5.43	2.03 - 2.00
Multiplicity	4.6	3.5	4.8
Completeness	59.56%	58.02%	60.69%
Mean I/σ(I)	10.7	26.4	2.4
Rmerge	0.169	0.105	0.497
Rmeas	0.192	0.126	0.559
Rpim	0.086	0.067	0.244
CC½	0.981	0.974	0.778
Rfree	0.260		
FSC average	0.949		
Thickness	212		

argon: grid 6; lamella 2			
	Overall	Low resolution	High resolution
Resolution (Å)	55.58 - 2.00	55.60 - 5.43	2.03 - 2.00
Multiplicity	3.7	2.8	3.8
Completeness	51.96%	51.86%	52.85%
Mean I/σ(I)	6.7	17.5	1.5
Rmerge	0.212	0.115	0.657
Rmeas	0.245	0.139	0.755
Rpim	0.117	0.075	0.352
CC½	0.974	0.983	0.604
Rfree	0.284		
FSC average	0.927		
Thickness	-		

argon: grid 6; lamella 4			
	Overall	Low resolution	High resolution
Resolution (Å)	55.70 - 2.00	55.72 - 5.43	2.03 - 2.00
Multiplicity	5.2	3.9	5.3
Completeness	46.89%	46.99%	46.90%
Mean I/σ(I)	9.7	25.2	2.1
Rmerge	0.189	0.103	0.631
Rmeas	0.211	0.121	0.700
Rpim	0.092	0.060	0.294
CC½	0.985	0.987	0.783
Rfree	0.308		
FSC average	0.919		
Thickness	135		

argon: grid 6; lamella 5			
	Overall	Low resolution	High resolution
Resolution (Å)	55.59 - 2.00	55.61 - 5.43	2.03 - 2.00
Multiplicity	4.4	3.3	4.4
Completeness	59.05%	57.91%	59.16%
Mean I/σ(I)	9.5	23.1	2.2
Rmerge	0.187	0.111	0.546
Rmeas	0.214	0.132	0.619
Rpim	0.099	0.069	0.280
CC½	0.978	0.981	0.720
Rfree	0.280		
FSC average	0.940		
Thickness	97		

argon: grid 6; lamella 6			
	Overall	Low resolution	High resolution
Resolution (Å)	55.65 - 2.00	55.67 - 5.43	2.03 - 2.00
Multiplicity	4.1	3.2	4.2
Completeness	57.17%	56.94%	57.95%
Mean I/σ(I)	5.0	13.6	0.9
Rmerge	0.272	0.129	1.045
Rmeas	0.313	0.152	1.187
Rpim	0.147	0.077	0.537
CC½	0.961	0.976	0.373
Rfree	0.338		
FSC average	0.897		
Thickness	152		

argon: grid 6; lamella 8			
	Overall	Low resolution	High resolution
Resolution (Å)	55.58 - 2.00	55.60 - 5.43	2.03 - 2.00
Multiplicity	4.5	3.3	4.3
Completeness	31.04%	32.37%	30.67%
Mean I/σ(I)	7.7	19.0	1.8
Rmerge	0.220	0.110	0.633
Rmeas	0.249	0.131	0.717
Rpim	0.113	0.069	0.327
CC½	0.982	0.989	0.611
Rfree	0.303		
FSC average	0.929		
Thickness	-		

argon: grid 7; lamella 1			
	Overall	Low resolution	High resolution
Resolution (Å)	55.45 - 2.00	55.47 - 5.43	2.03 - 2.00
Multiplicity	5.7	4.5	5.7
Completeness	93.48%	92.80%	95.07%
Mean I/σ(I)	8.0	23.0	1.3
Rmerge	0.229	0.106	1.244
Rmeas	0.255	0.121	1.390
Rpim	0.105	0.057	0.581
CC½	0.979	0.987	0.353
Rfree	0.258		
FSC average	0.948		
Thickness	172		

argon: grid 7; lamella 3			
	Overall	Low resolution	High resolution
Resolution (Å)	55.51 - 2.00	55.53 - 5.43	2.03 - 2.00
Multiplicity	5.6	4.2	5.7
Completeness	84.75%	83.33%	85.01%
Mean I/σ(I)	9.9	22.8	2.4
Rmerge	0.208	0.126	0.620
Rmeas	0.230	0.146	0.683
Rpim	0.095	0.069	0.278
CC½	0.981	0.973	0.718
Rfree	0.264		
FSC average	0.951		
Thickness	220		

argon: grid 7; lamella 4			
	Overall	Low resolution	High resolution
Resolution (Å)	55.57 - 2.00	55.59 - 5.43	2.03 - 2.00
Multiplicity	8.4	6.7	8.6
Completeness	68.84%	66.87%	70.02%
Mean I/σ(I)	8.8	25.2	1.5
Rmerge	0.253	0.125	1.216
Rmeas	0.270	0.136	1.298
Rpim	0.091	0.051	0.431
CC½	0.983	0.993	0.186
Rfree	0.284		
FSC average	0.935		
Thickness	98		

gallium: grid 8; lamella 1			
	Overall	Low resolution	High resolution
Resolution (Å)	55.94 - 2.00	55.96 - 5.43	2.03 - 2.00
Multiplicity	5.7	4.6	6.0
Completeness	89.87%	88.59%	88.76%
Mean I/σ(I)	9.5	24.7	1.9
Rmerge	0.208	0.113	0.709
Rmeas	0.230	0.129	0.777
Rpim	0.095	0.060	0.311
CC½	0.985	0.988	0.748
Rfree	0.259		
FSC average	0.946		
Thickness	189		

gallium: grid 8; lamella 2			
	Overall	Low resolution	High resolution
Resolution (Å)	55.80 - 2.00	55.83 - 5.43	2.03 - 2.00
Multiplicity	5.7	4.8	5.9
Completeness	90.40%	89.17%	90.80%
Mean I/σ(I)	3.1	13.7	0.2
Rmerge	0.430	0.141	4.385
Rmeas	0.477	0.160	4.839
Rpim	0.200	0.073	1.990
CC½	0.970	0.988	0.000
Rfree	0.306		
FSC average	0.886		
Thickness	171		

gallium: grid 8; lamella 3			
	Overall	Low resolution	High resolution
Resolution (Å)	55.48 - 2.00	55.50 - 5.43	2.03 - 2.00
Multiplicity	5.9	5.2	5.9
Completeness	88.49%	84.36%	88.70%
Mean I/σ(I)	2.9	15.8	0.2
Rmerge	0.508	0.131	4.430
Rmeas	0.558	0.146	4.861
Rpim	0.226	0.062	1.960
CC½	0.981	0.993	0.115
Rfree	0.337		
FSC average	0.871		
Thickness	-		

gallium: grid 8; lamella 4			
	Overall	Low resolution	High resolution
Resolution (Å)	55.70 - 2.00	55.72 - 5.43	2.03 - 2.00
Multiplicity	5.7	4.6	5.6
Completeness	94.44%	93.40%	93.92%
Mean I/σ(I)	8.6	24.4	1.3
Rmerge	0.206	0.105	0.920
Rmeas	0.228	0.120	1.017
Rpim	0.094	0.055	0.423
CC½	0.987	0.990	0.619
Rfree	0.254		
FSC average	0.949		
Thickness	216		

gallium: grid 9; lamella 1			
	Overall	Low resolution	High resolution
Resolution (Å)	55.58 - 2.00	55.60 - 5.43	2.03 - 2.00
Multiplicity	7.7	6.0	7.7
Completeness	77.14%	75.41%	77.51%
Mean I/σ(I)	12.7	32.3	2.5
Rmerge	0.183	0.102	0.623
Rmeas	0.196	0.113	0.666
Rpim	0.068	0.045	0.226
CC½	0.992	0.990	0.834
Rfree	0.264		
FSC average	0.952		
Thickness	159		

	gallium: grid 9; lamella 2		
	Overall	Low resolution	High resolution
Resolution (Å)	56.23 - 2.00	56.25 - 5.43	2.03 - 2.00
Multiplicity	5.9	4.7	5.9
Completeness	92.82%	91.81%	92.56%
Mean I/σ(I)	6.7	20.2	0.9
Rmerge	0.243	0.110	1.385
Rmeas	0.268	0.124	1.517
Rpim	0.109	0.055	0.609
CC½	0.986	0.992	0.432
Rfree	0.262		
FSC average	0.942		
Thickness	125		

	gallium: grid 9; lamella 3		
	Overall	Low resolution	High resolution
Resolution (Å)	55.54 - 2.00	55.57 - 5.43	2.03 - 2.00
Multiplicity	6.6	5.2	6.7
Completeness	79.36%	78.03%	78.77%
Mean I/σ(I)	11.0	27.4	2.1
Rmerge	0.180	0.097	0.630
Rmeas	0.196	0.109	0.682
Rpim	0.074	0.046	0.251
CC½	0.991	0.991	0.790
Rfree	0.264		
FSC average	0.951		
Thickness	124		

	gallium: grid 9; lamella 4		
	Overall	Low resolution	High resolution
Resolution (Å)	55.51 - 2.00	55.53 - 5.43	2.03 - 2.00
Multiplicity	6.0	4.8	5.8
Completeness	82.22%	80.17%	82.92%
Mean I/σ(I)	8.9	23.3	1.6
Rmerge	0.207	0.120	0.753
Rmeas	0.227	0.135	0.828
Rpim	0.088	0.058	0.324
CC½	0.986	0.988	0.680
Rfree	0.261		
FSC average	0.950		
Thickness	138		

gallium: grid 9; lamella 5			
	Overall	Low resolution	High resolution
Resolution (Å)	55.63 - 2.00	55.65 - 5.43	2.03 - 2.00
Multiplicity	5.8	4.6	5.8
Completeness	92.18%	91.15%	93.00%
Mean I/σ(I)	10.6	27.1	2.1
Rmerge	0.174	0.097	0.640
Rmeas	0.192	0.110	0.703
Rpim	0.079	0.050	0.284
CC½	0.989	0.992	0.753
Rfree	0.261		
FSC average	0.950		
Thickness	164		

gallium: grid 9; lamella 6			
	Overall	Low resolution	High resolution
Resolution (Å)	55.46 - 2.00	55.48 - 5.43	2.03 - 2.00
Multiplicity	5.9	4.8	6.0
Completeness	91.96%	90.74%	92.48%
Mean I/σ(I)	4.1	14.2	0.5
Rmerge	0.366	0.131	2.916
Rmeas	0.404	0.148	3.212
Rpim	0.166	0.066	1.303
CC½	0.973	0.991	0.124
Rfree	0.288		
FSC average	0.922		
Thickness	85		

gallium: grid 10; lamella 1			
	Overall	Low resolution	High resolution
Resolution (Å)	55.17 - 2.00	55.20 - 5.43	2.03 - 2.00
Multiplicity	6.0	4.8	6.1
Completeness	91.47%	89.92%	92.45%
Mean I/σ(I)	9.7	27.2	1.4
Rmerge	0.184	0.101	0.830
Rmeas	0.203	0.115	0.912
Rpim	0.081	0.052	0.364
CC½	0.989	0.992	0.725
Rfree	0.252		
FSC average	0.953		
Thickness	146		

gallium: grid 10; lamella 2			
	Overall	Low resolution	High resolution
Resolution (Å)	55.60 - 2.00	55.62 - 5.43	2.03 - 2.00
Multiplicity	7.2	5.7	7.4
Completeness	72.78%	71.22%	73.46%
Mean I/σ(I)	10.9	30.4	1.8
Rmerge	0.186	0.101	0.761
Rmeas	0.201	0.113	0.815
Rpim	0.072	0.046	0.281
CC½	0.991	0.990	0.808
Rfree	0.268		
FSC average	0.951		
Thickness	201		

gallium: grid 10; lamella 3			
	Overall	Low resolution	High resolution
Resolution (Å)	55.67 - 2.00	55.70 - 5.43	2.03 - 2.00
Multiplicity	6.1	4.9	6.3
Completeness	83.62%	82.18%	83.54%
Mean I/σ(I)	6.3	22.3	0.7
Rmerge	0.239	0.109	1.515
Rmeas	0.262	0.123	1.656
Rpim	0.103	0.054	0.636
CC½	0.987	0.991	0.211
Rfree	0.273		
FSC average	0.922		
Thickness	158		

gallium: grid 11; lamella 1			
	Overall	Low resolution	High resolution
Resolution (Å)	55.74 - 2.00	55.77 - 5.43	2.03 - 2.00
Multiplicity	4.4	3.5	4.4
Completeness	97.83%	97.30%	97.54%
Mean I/σ(I)	4.8	13.4	0.8
Rmerge	0.293	0.126	1.243
Rmeas	0.331	0.148	1.400
Rpim	0.150	0.074	0.626
CC½	0.968	0.987	0.428
Rfree	0.286		
FSC average	0.921		
Thickness	147		

gallium: grid 11; lamella 2			
	Overall	Low resolution	High resolution
Resolution (Å)	55.61 - 2.00	55.63 - 5.43	2.03 - 2.00
Multiplicity	6.6	5.3	6.7
Completeness	88.74%	87.24%	89.41%
Mean I/σ(I)	8.1	23.9	1.2
Rmerge	0.233	0.102	1.281
Rmeas	0.254	0.113	1.388
Rpim	0.094	0.046	0.506
CC½	0.988	0.991	0.476
Rfree	0.265		
FSC average	0.945		
Thickness	110		

gallium: grid 12; lamella 2			
	Overall	Low resolution	High resolution
Resolution (Å)	55.64 - 2.00	55.66 - 5.43	2.03 - 2.00
Multiplicity	6.0	4.8	6.2
Completeness	94.15%	93.20%	94.58%
Mean I/σ(I)	6.3	23.3	0.6
Rmerge	0.233	0.107	1.917
Rmeas	0.256	0.121	2.103
Rpim	0.103	0.054	0.837
CC½	0.988	0.991	0.126
Rfree	0.262		
FSC average	0.928		
Thickness	-		

gallium: grid 12; lamella 3			
	Overall	Low resolution	High resolution
Resolution (Å)	55.40 - 2.00	55.42 - 5.43	2.03 - 2.00
Multiplicity	6.3	5.0	6.3
Completeness	92.11%	90.53%	92.51%
Mean I/σ(I)	6.0	21.8	0.7
Rmerge	0.245	0.100	1.871
Rmeas	0.268	0.113	2.048
Rpim	0.104	0.049	0.797
CC½	0.987	0.991	0.224
Rfree	0.288		
FSC average	0.923		
Thickness	-		

gallium: grid 12; lamella 4			
	Overall	Low resolution	High resolution
Resolution (Å)	55.91 - 2.00	55.93 - 5.43	2.03 - 2.00
Multiplicity	4.6	3.7	4.7
Completeness	86.59%	85.05%	86.03%
Mean I/σ(I)	5.0	17.4	0.6
Rmerge	0.262	0.110	2.170
Rmeas	0.295	0.127	2.429
Rpim	0.127	0.062	1.032
CC½	0.982	0.986	0.109
Rfree	0.301		
FSC average	0.908		
Thickness	-		

gallium: grid 12; lamella 5			
	Overall	Low resolution	High resolution
Resolution (Å)	55.58 - 2.00	55.60 - 5.43	2.03 - 2.00
Multiplicity	3.4	2.7	3.5
Completeness	94.32%	93.42%	94.90%
Mean I/σ(I)	3.0	11.7	0.2
Rmerge	0.411	0.139	5.030
Rmeas	0.478	0.170	5.816
Rpim	0.236	0.095	2.832
CC½	0.960	0.979	-0.041
Rfree	0.317		
FSC average	0.887		
Thickness	-		

xenon: grid 13; lamella 1			
	Overall	Low resolution	High resolution
Resolution (Å)	56.04 - 2.00	56.06 - 5.43	2.03 - 2.00
Multiplicity	6.0	4.8	6.3
Completeness	87.69%	85.86%	86.59%
Mean I/σ(I)	11.1	28.3	2.3
Rmerge	0.166	0.088	0.612
Rmeas	0.182	0.099	0.668
Rpim	0.072	0.045	0.261
CC½	0.992	0.993	0.759
Rfree	0.251		
FSC average	0.953		
Thickness	-		

xenon: grid 13; lamella 2			
	Overall	Low resolution	High resolution
Resolution (Å)	55.67 - 2.00	55.69 - 5.43	2.03 - 2.00
Multiplicity	6.8	5.5	6.9
Completeness	75.57%	74.03%	75.00%
Mean I/σ(I)	9.9	27.0	1.8
Rmerge	0.180	0.098	0.729
Rmeas	0.195	0.109	0.787
Rpim	0.071	0.045	0.285
CC½	0.992	0.991	0.723
Rfree	0.272		
FSC average	0.950		
Thickness	-		

xenon: grid 13; lamella 3			
	Overall	Low resolution	High resolution
Resolution (Å)	55.90 - 2.00	55.92 - 5.43	2.03 - 2.00
Multiplicity	6.0	4.7	6.4
Completeness	90.91%	89.17%	90.34%
Mean I/σ(I)	11.1	28.5	2.4
Rmerge	0.167	0.091	0.617
Rmeas	0.184	0.103	0.673
Rpim	0.074	0.047	0.264
CC½	0.991	0.993	0.820
Rfree	0.256		
FSC average	0.954		
Thickness	-		

xenon: grid 13; lamella 4			
	Overall	Low resolution	High resolution
Resolution (Å)	55.65 - 2.00	55.67 - 5.43	2.03 - 2.00
Multiplicity	8.6	6.7	9.0
Completeness	69.20%	67.83%	69.80%
Mean I/σ(I)	13.7	33.6	3.1
Rmerge	0.178	0.106	0.611
Rmeas	0.190	0.115	0.648
Rpim	0.062	0.043	0.205
CC½	0.993	0.992	0.779
Rfree	0.263		
FSC average	0.956		
Thickness	-		

xenon: grid 13; lamella 5			
	Overall	Low resolution	High resolution
Resolution (Å)	56.93 - 2.00	56.95 - 5.43	2.03 - 2.00
Multiplicity	6.1	4.8	6.4
Completeness	95.47%	94.09%	95.78%
Mean I/σ(I)	9.6	26.2	1.6
Rmerge	0.187	0.095	0.915
Rmeas	0.205	0.107	1.000
Rpim	0.082	0.048	0.397
CC½	0.990	0.992	0.633
Rfree	0.279		
FSC average	0.942		
Thickness	-		

xenon: grid 13; lamella 6			
	Overall	Low resolution	High resolution
Resolution (Å)	78.30 - 2.00	78.36 - 5.43	2.03 - 2.00
Multiplicity	5.7	4.3	5.8
Completeness	100.00%	100.00%	99.75%
Mean I/σ(I)	9.4	23.6	2.0
Rmerge	0.181	0.100	0.694
Rmeas	0.200	0.114	0.762
Rpim	0.081	0.053	0.303
CC½	0.990	0.988	0.774
Rfree	0.256		
FSC average	0.952		
Thickness	-		

xenon: grid 13; lamella 7			
	Overall	Low resolution	High resolution
Resolution (Å)	55.54 - 2.00	55.56 - 5.43	2.03 - 2.00
Multiplicity	8.6	6.6	8.7
Completeness	65.60%	64.54%	66.92%
Mean I/σ(I)	13.6	32.2	3.2
Rmerge	0.178	0.106	0.569
Rmeas	0.188	0.115	0.604
Rpim	0.061	0.043	0.194
CC½	0.994	0.993	0.803
Rfree	0.271		
FSC average	0.949		
Thickness	-		

xenon: grid 13; lamella 8			
	Overall	Low resolution	High resolution
Resolution (Å)	55.41 - 2.00	55.43 - 5.43	2.03 - 2.00
Multiplicity	6.5	5.0	6.7
Completeness	89.82%	85.80%	90.41%
Mean I/σ(I)	11.4	29.5	2.2
Rmerge	0.171	0.092	0.642
Rmeas	0.186	0.104	0.696
Rpim	0.073	0.047	0.265
CC½	0.992	0.992	0.827
Rfree	0.247		
FSC average	0.956		
Thickness	-		

xenon: grid 13; lamella 9			
	Overall	Low resolution	High resolution
Resolution (Å)	55.43 - 2.00	55.46 - 5.43	2.03 - 2.00
Multiplicity	6.5	5.0	6.6
Completeness	89.71%	88.38%	90.25%
Mean I/σ(I)	10.8	27.1	2.3
Rmerge	0.174	0.098	0.672
Rmeas	0.190	0.111	0.731
Rpim	0.073	0.049	0.277
CC½	0.990	0.988	0.772
Rfree	0.266		
FSC average	0.945		
Thickness	-		

xenon: grid 13; lamella 10			
	Overall	Low resolution	High resolution
Resolution (Å)	37.45 - 2.00	37.46 - 5.42	2.03 - 2.00
Multiplicity	8.9	7.2	9.2
Completeness	65.26%	61.70%	67.16%
Mean I/σ(I)	13.5	32.5	3.3
Rmerge	0.194	0.116	0.612
Rmeas	0.206	0.125	0.646
Rpim	0.067	0.046	0.200
CC½	0.992	0.990	0.854
Rfree	0.264		
FSC average	0.952		
Thickness	-		

argon: grid 14; lamella 2			
	Overall	Low resolution	High resolution
Resolution (Å)	55.88 - 2.00	55.91 - 5.43	2.03 - 2.00
Multiplicity	6.3	5.0	6.5
Completeness	81.59%	79.21%	80.98%
Mean I/σ(I)	11.1	29.4	2.3
Rmerge	0.182	0.098	0.679
Rmeas	0.199	0.110	0.737
Rpim	0.079	0.049	0.282
CC½	0.989	0.991	0.825
Rfree	0.252		
FSC average	0.951		
Thickness	-		

argon: grid 14; lamella 3			
	Overall	Low resolution	High resolution
Resolution (Å)	37.52 - 2.00	37.52 - 5.42	2.03 - 2.00
Multiplicity	8.2	6.6	8.4
Completeness	68.48%	66.12%	70.27%
Mean I/σ(I)	8.4	23.5	2.0
Rmerge	0.276	0.147	1.070
Rmeas	0.294	0.159	1.147
Rpim	0.097	0.059	0.384
CC½	0.985	0.989	0.597
Rfree	0.275		
FSC average	0.941		
Thickness	-		

argon: grid 14; lamella 4			
	Overall	Low resolution	High resolution
Resolution (Å)	55.61 - 2.00	55.63 - 5.43	2.03 - 2.00
Multiplicity	5.8	4.5	6.0
Completeness	97.75%	97.32%	97.54%
Mean I/σ(I)	8.8	23.3	1.7
Rmerge	0.208	0.115	0.840
Rmeas	0.229	0.131	0.919
Rpim	0.093	0.060	0.361
CC½	0.984	0.975	0.665
Rfree	0.265		
FSC average	0.949		
Thickness	-		

argon: grid 14; lamella 5			
	Overall	Low resolution	High resolution
Resolution (Å)	55.74 - 2.00	55.76 - 5.43	2.03 - 2.00
Multiplicity	5.9	4.5	6.1
Completeness	96.92%	96.27%	97.08%
Mean I/σ(I)	10.3	26.8	2.0
Rmerge	0.189	0.097	0.742
Rmeas	0.207	0.110	0.810
Rpim	0.083	0.049	0.319
CC½	0.990	0.993	0.794
Rfree	0.238		
FSC average	0.955		
Thickness	-		

argon: grid 14; lamella 7			
	Overall	Low resolution	High resolution
Resolution (Å)	55.54 - 2.00	55.56 - 5.43	2.03 - 2.00
Multiplicity	5.3	4.2	5.4
Completeness	92.07%	91.15%	92.79%
Mean I/σ(I)	5.1	15.0	0.8
Rmerge	0.288	0.135	1.473
Rmeas	0.320	0.154	1.635
Rpim	0.132	0.070	0.670
CC½	0.972	0.982	0.226
Rfree	0.276		
FSC average	0.933		
Thickness	-		

gallium: grid 15; lamella 1			
	Overall	Low resolution	High resolution
Resolution (Å)	55.55 - 2.00	55.57 - 5.43	2.03 - 2.00
Multiplicity	6.9	5.4	7.0
Completeness	76.95%	75.10%	76.70%
Mean I/σ(I)	9.0	27.8	1.2
Rmerge	0.216	0.108	1.232
Rmeas	0.233	0.120	1.324
Rpim	0.084	0.049	0.461
CC½	0.990	0.989	0.522
Rfree	0.266		
FSC average	0.945		
Thickness	-		

gallium: grid 15; lamella 2			
	Overall	Low resolution	High resolution
Resolution (Å)	55.60 - 2.00	55.62 - 5.43	2.03 - 2.00
Multiplicity	6.2	5.0	6.5
Completeness	84.50%	83.09%	84.67%
Mean I/σ(I)	6.4	20.9	0.8
Rmerge	0.251	0.105	1.741
Rmeas	0.275	0.118	1.893
Rpim	0.106	0.052	0.706
CC½	0.986	0.989	0.292
Rfree	0.284		
FSC average	0.930		
Thickness	-		

gallium: grid 15; lamella 3			
	Overall	Low resolution	High resolution
Resolution (Å)	55.52 - 2.00	55.54 - 5.43	2.03 - 2.00
Multiplicity	5.8	4.8	6.1
Completeness	90.32%	87.45%	91.69%
Mean I/σ(I)	2.9	11.1	0.3
Rmerge	0.664	0.196	3.959
Rmeas	0.728	0.221	4.306
Rpim	0.290	0.098	1.665
CC½	0.927	0.988	0.103
Rfree	0.372		
FSC average	0.863		
Thickness	-		

gallium: grid 15; lamella 4			
	Overall	Low resolution	High resolution
Resolution (Å)	55.63 - 2.00	55.65 - 5.43	2.03 - 2.00
Multiplicity	7.0	5.5	7.3
Completeness	77.16%	75.31%	76.96%
Mean I/σ(I)	8.5	26.3	1.2
Rmerge	0.212	0.107	1.197
Rmeas	0.229	0.119	1.288
Rpim	0.084	0.049	0.453
CC½	0.990	0.992	0.479
Rfree	0.266		
FSC average	0.949		
Thickness	-		

gallium: grid 15; lamella 5			
	Overall	Low resolution	High resolution
Resolution (Å)	55.47 - 2.00	55.49 - 5.43	2.03 - 2.00
Multiplicity	6.3	5.0	6.6
Completeness	84.45%	80.04%	84.84%
Mean I/σ(I)	5.3	16.8	0.8
Rmerge	0.307	0.123	1.554
Rmeas	0.335	0.138	1.689
Rpim	0.132	0.061	0.654
CC½	0.981	0.990	0.295
Rfree	0.277		
FSC average	0.934		
Thickness	-		

gallium: grid 15; lamella 6			
	Overall	Low resolution	High resolution
Resolution (Å)	55.63 - 2.00	55.65 - 5.43	2.03 - 2.00
Multiplicity	5.8	4.5	6.0
Completeness	94.39%	93.42%	94.75%
Mean I/σ(I)	8.9	24.9	1.4
Rmerge	0.201	0.106	0.949
Rmeas	0.222	0.122	1.039
Rpim	0.090	0.057	0.407
CC½	0.984	0.976	0.508
Rfree	0.254		
FSC average	0.949		
Thickness	-		

gallium: grid 15; lamella 9			
	Overall	Low resolution	High resolution
Resolution (Å)	55.69 - 2.00	55.71 - 5.43	2.03 - 2.00
Multiplicity	6.3	4.9	6.4
Completeness	91.55%	90.44%	92.15%
Mean I/σ(I)	6.1	19.8	0.7
Rmerge	0.263	0.121	1.678
Rmeas	0.288	0.137	1.833
Rpim	0.112	0.061	0.701
CC½	0.982	0.983	0.096
Rfree	0.276		
FSC average	0.933		
Thickness	-		

gallium: grid 15; lamella 10			
	Overall	Low resolution	High resolution
Resolution (Å)	37.76 - 2.00	37.77 - 5.42	2.03 - 2.00
Multiplicity	5.9	4.8	5.9
Completeness	69.01%	65.02%	70.44%
Mean I/σ(I)	4.5	13.5	0.8
Rmerge	0.402	0.181	1.860
Rmeas	0.438	0.203	2.016
Rpim	0.166	0.088	0.739
CC½	0.963	0.970	0.268
Rfree	0.272		
FSC average	0.931		
Thickness	-		

argon: grid 16; lamella 1			
	Overall	Low resolution	High resolution
Resolution (Å)	55.77 - 2.00	55.79 - 5.43	2.03 - 2.00
Multiplicity	5.8	4.8	5.9
Completeness	95.47%	94.79%	95.37%
Mean I/σ(I)	3.9	17.6	0.3
Rmerge	0.433	0.126	6.230
Rmeas	0.479	0.142	6.883
Rpim	0.198	0.063	2.848
CC½	0.971	0.988	-0.070
Rfree	0.296		
FSC average	0.898		
Thickness	-		

argon: grid 16; lamella 4			
	Overall	Low resolution	High resolution
Resolution (Å)	55.52 - 2.00	55.54 - 5.43	2.03 - 2.00
Multiplicity	6.5	5.4	6.5
Completeness	82.43%	80.79%	83.00%
Mean I/σ(I)	4.5	19.6	0.4
Rmerge	0.380	0.121	3.338
Rmeas	0.413	0.133	3.612
Rpim	0.152	0.053	1.307
CC½	0.983	0.991	-0.000
Rfree	0.273		
FSC average	0.907		
Thickness	-		

argon: grid 16; lamella 5			
	Overall	Low resolution	High resolution
Resolution (Å)	55.64 - 2.00	55.66 - 5.43	2.03 - 2.00
Multiplicity	6.7	5.5	6.8
Completeness	73.18%	71.01%	73.58%
Mean I/σ(I)	6.9	23.4	0.9
Rmerge	0.310	0.124	2.211
Rmeas	0.335	0.137	2.364
Rpim	0.120	0.056	0.801
CC½	0.982	0.965	0.384
Rfree	0.296		
FSC average	0.918		
Thickness	211		
